# Endothelial Dysfunction as a Key Link between Cardiovascular Disease and Frailty: A Systematic Review

**DOI:** 10.3390/jcm13092686

**Published:** 2024-05-02

**Authors:** Hakan Calila, Elena Bălășescu, Roxana Ioana Nedelcu, Daniela Adriana Ion

**Affiliations:** 1 Laboratory of Experimental Medicine and Fundamental Research, 2nd Pathophysiology Department, Carol Davila University of Medicine and Pharmacy, 37 Dionisie Lupu Street, District 2, 020021 Bucharest, Romania; roxana.nedelcu@umfcd.ro (R.I.N.); daniela.ion@umfcd.ro (D.A.I.); 2 SanacareVital Clinic, 010161 Bucharest, Romania

**Keywords:** frailty, cardiovascular diseases, vascular endothelial dysfunction, arterial stiffness, pathophysiology

## Abstract

**Background:** Frailty is increasingly recognized as a significant health concern, particularly due to its association with cardiovascular pathologies. This study aims to examine how vascular endothelial dysfunction, a known premorbid stage in the pathophysiology of cardiovascular diseases, contributes to the link between cardiovascular illness and frailty. **Methods:** The inclusion criteria allowed us to focus on original clinical research articles published in English between January 2014 and January 2024, which reported quantitative assessments of the relationship between frailty and vascular endothelial dysfunction. Excluded from the study were systematic literature reviews, meta-analyses, editorials, conference articles, theses, methodological articles, and studies using animal or cell culture models. Searches were conducted of electronic databases, including Scopus, ScienceDirect, and Medline, up to 22 January 2024. The risk of bias was assessed using the Joanna Briggs Institute’s critical appraisal tools. The methods used to present and synthesize the results involved data extraction and categorization based on biomolecular and clinical findings of endothelial dysfunction. **Results:** Following the application of the inclusion and exclusion criteria, a total of 29 studies were identified. Vascular endothelial dysfunction was associated with increased frailty phenotypes, and we also identified SGLT-2 inhibitors’ potential role as an anti-fragility treatment that affects endothelial dysfunction. This study found that the physical and biomolecular markers of endothelial dysfunction are associated with frailty measures and have predictive value for incident frailty. Furthermore, some studies have shown inflammation to have an impact on endothelial dysfunction and frailty, and an innovative age-related chronic inflammation measure has been proven to predict frailty scores. **Conclusions:** The current evidence suggests an association between endothelial dysfunction and frailty, highlighting the need for further research to elucidate the underlying mechanisms.

## 1. Introduction

Frailty is an emerging public health problem with a well-defined presence among the elderly population. Defined as a state of vulnerability arising as a result of degradation of the physiological reserves and the capacity to uphold homeostasis, potentially leading to diminished resilience against stressors [1,2], frailty can be defined according to two models: the Fried phenotype model and the frailty index [2,3,4]. The Fried phenotype model characterizes frailty based on three out of five components: low grip strength, reduced walking speed, reduced physical activity or decreased mobility, increased fatigue, and unintentional weight loss [1,2]. The frailty index investigates the accumulation of deficits at the level of different systems [3].

Given the high prevalence of cardiovascular pathologies in the elderly population [5], frailty is an important prognostic factor for this demographic group [6,7]. The most important cardiovascular risk factors (high blood lipid levels, hypertension, diabetes, obesity, family history of coronary heart disease) are prevalent among the elderly [8,9]. Although the relationship between cardiovascular disease (CVD) and frailty has been established, the pathophysiological processes underlying the link between these two phenomena remain largely unexplained. It has been suggested that CVD and frailty may share a number of common pathophysiological processes [10]. In this respect, endothelial dysfunction appears to play an important role as a connection between CVD and frailty [10].

Endothelial dysfunction has been identified as an early, subclinical stage of CVD, suggesting its role in cardiovascular pathologies such as atherosclerosis, acute coronary syndromes, hypertension, heart failure, and aging. The latter is associated with increased inflammatory status, which has been documented in both frailty and endothelial dysfunction. In frail people, it has been described as a compromised, dysregulated complex dynamic system of the body due to chronic, low-grade inflammation and an altered energy metabolism [11]. Oxidative stress and the decreased availability of nitric oxide (NO), an endogenous vasodilator produced by the endothelium, were reported to be key elements in the impairment of endothelial function observed with advancing age [12]. At the same time, the deregulation of circuits involving miRNAs was revealed to play an important and emerging role in the development of endothelial dysfunction, with miRNAs playing a key part in the control of endothelial cell function [13].

It is important to characterise the association between the early stages of CVD and frailty because early detection and management strategies are essential for improving the quality of life of older people. At the same time, the implementation of early interventions to prevent the progression of frailty leads to reductions in the healthcare costs associated with hospital admissions and re-admissions related to the effects of frailty. Significant in this regard is the systematic review recently published by A. T. Amaraseskera and colleagues [10], in which the possible pathophysiological links between frailty, sarcopenia, and endothelial dysfunction are addressed. However, in recent years, the number of articles addressing the possible mechanisms involved in the link between endothelial dysfunction and frailty has increased significantly. Thus, this systematic review aimed to highlight the possible associations between inflammatory changes at the vascular level and physical frailty, as well as reviewing their underlying pathophysiological mechanisms.

## 2. Materials and Methods

### 2.1. Systematic Review Methods

This systematic review was conducted in accordance with the Preferred Reporting Items of Systematic Reviews and Meta-analysis (PRISMA Guidelines) [14], and the PRISMA 2020 checklist was used to structure and record the review items (Table S1 and Supplementary Chapter S1 in the Supplementary Materials).

### 2.2. Search Strategy

We conducted a systematic online search of medical databases, including Scopus, ScienceDirect, and Medline, considering all possible associations between frailty and all entities associated with endothelial dysfunction/subclinical cardiovascular disease. After the initial search, a chronology covering only the last 10 years was selected to capture all of the relevant and recent literature available, and to identify new published articles in relation to the systemic review of A. T. Amarasekera et al. [10]. The search terms and key search strategies are listed in Table 1. The latter were used to identify all the articles that contained these search phrases within the title/abstract/keywords. Supplementary Chapter S1: Systematic review methods contains full details of the literature search strategy, study selection criteria, data extraction and analysis, and quality assessments of the included studies. Additionally, principles for discussing non-randomised designs can be found in the Transparent Reporting of Non-randomised Designs (TREND) declaration [15].

### 2.3. Selection Criteria

All original clinical research studies that assessed the association between vascular endothelial dysfunction and frailty in a clinical setting were included in the systematic review. A quantitative assessment of the relationship between frailty and subclinical cardiovascular disease/vascular endothelial dysfunction in the clinical setting had to be reported by the article for inclusion.

A three-stage search strategy was used. An initial limited search was conducted of PMC (PubMed Central) and Google Scholar, followed by an analysis of the words contained in the titles and abstracts and the indexing terms used to describe the identified articles. A second search, using all of the identified keywords and indexing terms (Table 2), was performed using all of the included databases. Finally, reference lists of the relevant identified articles were manually searched for additional relevant studies. Following the search, duplicates were eliminated. Subsequently, after applying the inclusion and exclusion criteria described above, we analysed the full texts of the remaining articles and identified the 29 articles included in the current study.

### 2.4. Data Extraction and Synthesis Methods

Data including the first author, year of publication, reference, study design, participant characteristics and age (if available), sample size, sample stratification (if available), vascular endothelial dysfunction and physical frailty outcome measures, results, and author conclusions were extracted and evaluated (Table S2). 

### 2.5. Quality Assessment of the Studies Selected for Review

The 29 selected articles were assessed for methodological quality using the Joanna Briggs Institute’s critical appraisal tools [16]. Thus, the JBI critical appraisal tool for cross-sectional studies was used to assess the 26 articles identified in this category, and the JBI critical appraisal tool for cohort studies was used for 3 of the 29 articles included in this review (Table S3).

### 2.6. Certainty Assessment

This review used a qualitative method based on the GRADE (Grading of Recommendations, Assessment, Development and Evaluation) principles [17] to assess the certainty of the evidence for endothelial dysfunction, focusing on the overall quality of evidence from all of the included studies. The assessment considered methodological rigor, consistency, and the strength of the correlation between endothelial dysfunction and frailty in the body of evidence.

## 3. Results

### 3.1. Characteristics of the Included Studies

**Figure 1:**
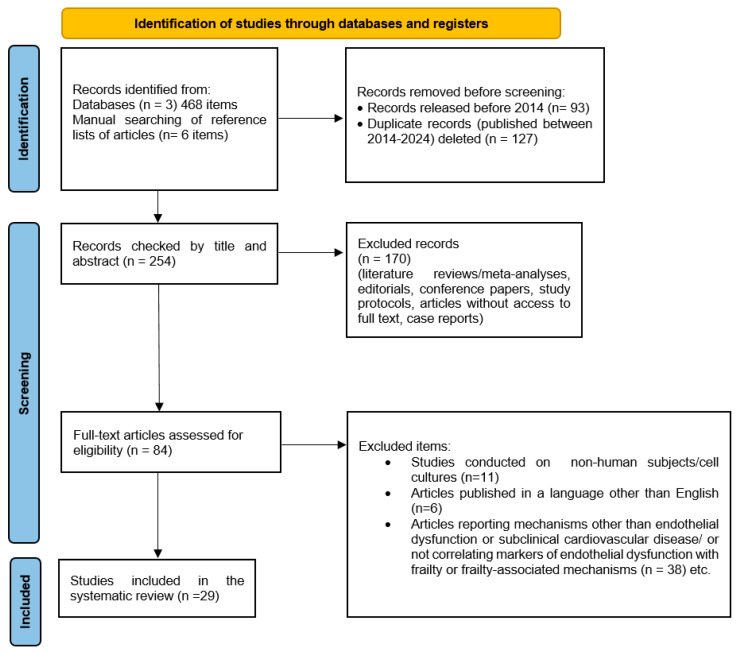
Flowchart of inclusion criteria.

A flow chart of the data processed at different stages of the analysis is shown in Figure 1.

A summary of the characteristics of the selected studies is presented in Table S2. The included studies were published between 2014 and 2024. The studies were conducted in Brazil (*n* = 2 [18,19]), China (*n* = 3 [20,21,22]), Egypt (*n* = 1 [23]), France (*n* = 2 [24,25]), India (*n* = 1 [26]), Japan (*n* = 2 [26,27]), Iceland (*n* = 1 [28]), Italy (*n* = 3 [29,30,31]), Korea (*n* = 3 [32,33,34]), the Netherlands (*n* = 1 [35]), the United Kingdom (*n* = 2 [36,37]), Singapore (*n* = 1 [38]), Spain (*n* = 4 [39,40,41,42]), and the United States of America (*n* = 4 [43,44,45,46]). All of the studies that were selected were conducted in clinical settings; the included works consisted primarily of studies with a descriptive cross-sectional design. The inclusion and exclusion criteria and sample sizes were reported in most of the included studies. The sample sizes of the included clinical trials ranged from 40 to 5764. Of the studies that were excluded, 11 were conducted in animal models and/or cell cultures. Of these, two studies presented information relevant to the context of the current study.

In a study conducted by Justice and collaborators [47], sodium nitrite supplementation in old male mice showed significant improvements in motor function and endurance compared to young mice. Supplementation ameliorated chronic low-grade inflammation in skeletal muscle, while highlighting the potential role of nitrite in increasing the bioavailability of nitric oxide (NO), which could improve blood flow and vascularization, contributing to systemic function. These findings suggest that sodium nitrite supplementation may be effective in preventing or treating the motor dysfunction associated with primary aging in humans, highlighting the role of nitric oxide in this context. On the other hand, Malavolta et al. [48] investigated the relationship between the BPIFB4 gene haplotype and frailty, finding that individuals with the LAV haplotype had a lower prevalence of frailty and higher survival rates compared to those with the RV haplotype. Gene therapy using the LAV-BPIFB4 gene attenuated clinical frailty in aged mice. Overall, the study highlighted the role of endothelial dysfunction and inflammation in frailty and suggested that LAV-BPIFB4 gene therapy may counteract these processes.

In addressing the validity of the studies, it became apparent that a critical issue was the lack of documented methods for adjusting for potential confounders. For instance, in a study conducted by Kannegieter [35] et al. on the association between frailty and mobility, handgrip strength, and aortic stiffness, although possible confounders were identified, no specific methods were applied to address them, such as matching or stratifying participants or utilizing multivariate regression analysis. This limitation was also observed in similar studies conducted by Macedo [18] and Mone [29].

### 3.2. Measurement of the Study Results

The methods used to measure vascular endothelial function and physical fragility were inconsistent across the included studies, with some studies using multiple measures. Of the 29 studies included in the review, carotid intima–media thickness (CIMT; *n* = 7 [22,26,28,33,36,37,38]), the ankle–brachial pressure index (ABPI; *n* = 4 [22,36,37,45]), carotid–femoral pulse wave velocity (cfPWV; *n* = 7 [25,36,37,38,41,44,45]), ankle–brachial pulse wave velocity (baPWV; *n* = 4 [20,21,26,32]), carotid distensibility (CD; *n* = 3 [36,37,38]), nitrite/nitric oxide (NO; *n* = 2 [23,31]), flow-mediated brachial dilatation (FMD; *n* = 2 [19,32]), the reactive hyperaemia index (RHI; *n* = 2 [34,38]), blood pressure complexity (*n* = 2 [20,21]), the heart–ankle vascular index (CAVI; *n* = 3 [22,26,27]), aortic pulse wave velocity (aPWV; *n* = 2 [18,35]), the aortic augmentation index (aAIx; *n* = 1 [38]), asymmetric dimethylarginine (ADMA; *n* = 1 [40]), homocysteine (Hcy; *n* = 2 [24,39]), the von Willebrand factor (vWF; *n* = 1 [36]), microRNA (miRNA; *n* = 1 [30]), the albumin–creatinine ratio (UACR; *n* = 1 [46]), the coronary calcium score (CAC; *n* = 1 [28]), aortic pulse pressure (aPP; *n* = 1 [38]), and chemokine CXCL9 (*n* = 1 [43]) were used as measures of vascular endothelial function.

Based on the articles included for the full-text review, many convincing data obtained via various biochemical and physiological parameters support the positive association between endothelial dysfunction or the subclinical state of CVD and physical frailty. The findings are summarized in Table S2.

### 3.3. Physiological Markers Linking Endothelial Dysfunction to Fragility

Over time, several physiological markers have been introduced into clinical practice that can assess arterial stiffness. One of these markers is flow-mediated vasodilation (FMD). It quantifies the ability of the endothelium to produce an NO-dependent response to shear stress and can thus be used as a direct measure of vascular endothelial function [49]. Mansur [19] et al. showed a relative frequency of patients with FMD ≥ 10% that was lower in a group of frail compared to non-frail patients among patients pre-diagnosed with stages 3–5 of chronic kidney disease. In a similar study, Park et al. [32] reported a similar association between frailty and reduced FMD, with significant associations observed even after adjusting for confounders.

In addition to FMD, the researchers used pulse wave velocity (PWV) to assess arterial stiffness, showing significantly higher values for this parameter in the pre-frail and frail groups compared to non-frail individuals. This marker is the most widely used measure of arterial stiffness. PWV is calculated by measuring the velocity of blood pressure waves travelling along the aorta and large arteries, usually by dividing the distance by the transit time of the pressure waves at the two points of arterial recording [50]. Other similar studies [18,20,21,26,36,38,41,44,45] support this observation, demonstrating a significant increase in PWV in frail or pre-frail patients and/or a correlation between a significant increase in PWV and one or more individual elements (e.g., grip force or gait velocity) belonging to the definition criteria of Fried’s frailty phenotype [51]. In addition, an adjusted analysis in another study [44] demonstrated a significant association between the frailty level and the carotid–femoral pulse wave velocity (cfPWV), further highlighting the role of arterial stiffness in frailty. However, a study of a cohort of 117 elderly patients conducted by Kannegieter [35] et al. failed to show a correlation between frailty and aortic stiffness quantified by aortic pulse wave velocity (aPWV). However, it is worth noting that the results obtained in this study were not adjusted for possible confounding variables, which could affect their validity.

As an alternative to pulse wave velocity, Papaioannou [25] et al. proposed the use of total arterial compliance as a surrogate marker of arterial stiffness. In a longitudinal study of an initial cohort of 279 older adults, they compared the prognostic value of PWV and total arterial compliance (TC). Arterial compliance is the ability of vessels to relax with increasing transmural pressure; thus, it is the opposite of arterial stiffness [25]. In this study, total arterial compliance emerged as a significant predictor of mortality and was also independently associated with age, highlighting its potential role in assessing the association between frailty and endothelial dysfunction.

The pathophysiological changes observed in early atherosclerosis are characterized by a decline in vascular endothelial function, which serves as a crucial initial phase in the development of atherosclerosis. Reactive hyperaemia peripheral arterial tonometry (RH-PAT) provides an automated and non-invasive means of measuring flow-induced arterial dilation mediated by endothelial cell function using a derived index called the reactive hyperaemia index (RHI) [52]. In a study conducted by Yoo et al. [34] on a cohort of 236 elderly women, an association between endothelial dysfunction, as estimated using RH-PAT, and grip force was observed, but this association has not been confirmed in other similar studies [38].

Another commonly used surrogate marker of arterial stiffness is carotid intima–media thickness (CIMT), which is a marker that can be determined via carotid ultrasound [53]. A study conducted by Park et al. [32] conducted on a cohort of 412 adults aged 70–88 years showed a positive correlation between CIMT and frailty, with CIMT being higher in this group of patients than in the non-frail group. This significant association was also supported by other similar studies [22,26,38], which have shown the association of CIMT with frailty or the constitutive elements of the Fried frailty phenotype (grip strength, gait speed, etc.) [51]. In a longitudinal descriptive study undertaken by Veronese et al. [5] on a cohort of 5764 participants belonging to the Reykiavik study, this association could not be demonstrated; however, according to the initial data, frail patients had more moderate or severe carotid plaques and a higher coronary calcium score. In the same study, it was observed that the cumulative incidence of cardiovascular disease was higher in frail patients. Notably, the coronary calcium score and presence of carotid plaques have better predictive value than CIMT for cardiovascular events [54].

The ankle–brachial index (ABPI) is a routinely determined parameter in patients with suspected peripheral arterial disease, and it is also a surrogate marker of atherosclerosis [2,55]. Nadruz et al. [45] analysed a sample of patients participating in the Atherosclerosis Risk in Communities (ARIC) study and found an association between abnormal values of both PWV and ABPI and frailty. In a similar study, Xue et al. [22] demonstrated a similar association between ABPI and frailty. An association between the heart–ankle vascular index (CAVI) and the constituents of the Fried phenotype (grip strength, gait speed, etc.) was also found.

CAVI is calculated using the heart-to-ankle PWV from the origin of the aortic valve to the ankle region and blood pressure measured at the arm; it is an index of global arterial stiffness [56]. Shiraishi et al. showed a significant correlation between CAVI and patients’ sedentary behaviour in a group of 116 Japanese patients with frailty [27]. Similar studies support the association between this marker and frailty; Yamanashi [26] et al. demonstrated a negative association between arterial stiffness (estimated using CAVI and PWV) and grip strength in non-hypertensive patients, and CAVI was identified as an independent risk factor for frailty in a study conducted by Xue et al. on 171 patients [22].

In addition to the markers listed above, one measure of the pathophysiological mechanisms that translate changes in cardiovascular regulation in the elderly appears to be blood pressure complexity (cxPA) [20]. BP complexity is quantified using multiscale entropy (MSE), which incorporates a coarse-grained process to assess the variability and irregularity of blood pressure patterns at different scales [20]. Two studies conducted by Jiang et al. found that lower systolic or diastolic BP complexity correlates with a higher chance of being pre-frail or frail [21]; they also demonstrated an inverse correlation between arterial stiffness and cxPA [20]. Thus, cxPA is a mediator between arterial stiffness and frailty.

Particularly important in assessing the association between various physiological markers of arterial stiffness and frailty are the contributions made by McKechnie et al. In addition to cross-sectional associations between markers of arterial stiffness and frailty [36], as demonstrated in a cross-sectional study of 1399 patients, they showed in two different studies the existence of a longitudinal association between subclinical CVD and incident frailty. In a group of 1057 red patients initially evaluated longitudinally in the British Regional Heart Study, the study found a higher risk of incident frailty in subjects with higher CIMT and PWV values [37].

Collective evidence highlights the complex relationship between vascular health and frailty among older adults. However, we emphasize the need for further studies of larger groups of patients that take into account possible confounders to support the correlation between arterial stiffness, as assessed by the physiological markers described above, and frailty. The findings are summarized in Table 3.

### 3.4. SGLT2 Inhibitor Treatment as a Potential Link between Frailty and Endothelial Dysfunction

In the context of improving endothelial function and lowering levels of certain microRNAs involved in age-related pathologies, our review included two interventional studies conducted by Mone et al. [29,30], which highlight the role of SGLT-2 inhibitors in delaying endothelial cell aging (Table 4). The first study [29] shows that empagliflozin improves cognitive impairment and physical decline, possibly by attenuating the mitochondrial calcium overload and reducing the production of reactive oxygen species (ROS) in endothelial cells. Given the known associations between endothelial dysfunction, oxidative stress, and frailty, these findings suggest that the beneficial effects of empagliflozin on physical and cognitive function may involve the attenuation of endothelial dysfunction and oxidative stress. The second study [30] reveals that treatment with empagliflozin leads to significant changes in levels of microRNAs (miRs) associated with endothelial disfunction. Specifically, miRNAs such as miR-21, miR-92, and miR-221 [13] are down-regulated after empagliflozin treatment in patients with heart failure with preserved ejection fraction (HFpEF) and diabetes mellitus (DM). These miRs are known to regulate angiogenesis, vascular function, and age-related diseases [13], suggesting that their down-regulation may reflect a restoration of endothelial function. Therefore, the effects of empagliflozin on cognitive and physical function, as well as the modulation of miRs associated with endothelial dysfunction, provide compelling evidence for a potential link between frailty and endothelial dysfunction, with empagliflozin acting as a therapeutic agent that simultaneously targets both conditions.

### 3.5. The Intersection of Inflammation, Cardiovascular Health, and Frailty: Homocysteine, C-Reactive Protein, and Other Immune Biomarkers

Inflammation plays a key role in both frailty and cardiovascular pathology. In this regard, the existence of chronic inflammatory status has been identified as one of the eight pillars of cardiovascular aging alongside disabled macroautophagy, a loss of proteostasis, genomic instability, epigenetic alterations, mitochondrial dysfunction, cell senescence, and dysregulated neurohormonal signalling [57].

Homocysteine (Hcy) and C-reactive protein (CRP) are molecules that play an important role in inflammation and cardiovascular health. At the same time, both molecules have the ability to modulate various pathways that lead to muscle wasting, decreased physical performance, and, ultimately, frailty [39].

In a study conducted by Álvarez-Sánchez [39] et al. on Spanish people aged 65 years or older, elevated levels of both Hcy and high-sensitivity C-reactive protein (hsCRP) were independently associated with frailty, with hsCRP also being associated with pre-frailty. Furthermore, these inflammatory biomarkers were correlated with components of the Fried frailty score [51], including exhaustion, frailty, and low physical activity. This suggests the crucial role of inflammation; this is particularly evident in age-related physical decline, which may underpin frailty. 

The association of homocysteine levels with frailty status has also been highlighted by other studies. In a study conducted by Guillotin et al. [24] on a cohort with suspected normal-pressure hydrocephalus (NPH), Hcy was highlighted as the only parameter independently associated with both the frailty index (FI) and central nervous system (CNS) biomechanical responses. This suggests a potential biological pathway linking frailty to CNS biomechanics, with Hcy playing a central role. These findings align with previous evidence indicating an association between inflammation, suggested by markers such as hsCRP, and frailty [58], while highlighting the unique contribution of Hcy to frailty and CNS alterations. Mechanistically, Hcy is implicated in neurodegeneration, neuroinflammation, and cardiovascular risk, suggesting multifaceted pathways by which it may influence both frailty and CNS function [39]. These insights highlight the importance of further investigations of Hcy as a potential therapeutic target in addressing frailty and its associated neurobiological changes.

The search for reliable biomarkers to identify and monitor frailty has gained momentum in recent years as researchers have sought out more accurate methods of early detection and intervention. In a recent study [42], immune biomarkers were assessed in the non-frail, pre-frail, and frail groups, highlighting their association with frailty status. Interestingly, while lymphocyte subset ratios showed limited associations with frailty severity, concentrations of key inflammatory molecules such as IL6, CRP, TNFα, and sTNF-RII showed notable deviations from baseline ranges in frail individuals, highlighting the potential role of chronic inflammation in frailty progression. In this context, sTNF-RII was identified as a promising candidate for frailty screening, showing strong associations with frailty status and demonstrating high predictive value in distinguishing frail subjects. However, we highlight the need for further research to elucidate the mechanisms underlying the link between inflammation and frailty, as well as validation of the findings in larger cohorts.

Given the increasing number of studies that point to a link between inflammatory markers and age or frailty, the possibility has arisen of constructing a measure of age based on the chronic inflammation that physiologically accompanies senescence. In this regard, Sayed et al. [43] conducted immune phenotyping of patients participating in the 1000 Immunomes project with the aim of constructing a measure for chronic age-related inflammation (iAge). The study identified immune biomarkers of aging and established baseline values for systemic chronic age-related inflammation. Using artificial intelligence, the researchers developed an ‘inflammatory clock’ of aging, taking into account the non-linear relationship and redundancy of the cytokine network. The study demonstrated the power of iAge to predict frailty scores based on immune biomarkers in the blood. The study also highlights CXCL9 as a significant player in cardiovascular pathology, independent of age, with endothelial cells playing a central role in cardiovascular aging. CXCL9 may exacerbate endothelial dysfunction with age, and its role and that of other CXC chemokines in cardiovascular pathology have been demonstrated in previous clinical studies [59].

The synthesis of our inquiries into diverse facets of inflammation is encapsulated within Table 4.

### 3.6. Alteration of NO Signalling in People with Frailty, and the Role of ADMA as a Potential Biomarker

Asymmetric dimethyl arginine (ADMA) has emerged as a potential biomarker of frailty in people without clinical cardiovascular disease, with previous studies demonstrating the role of ADMA as a marker of endothelial dysfunction and as a predictor of CVD risk and mortality [60] (Table 4). A study carried out by Alonzo Bouson [40] et al. adds to this body of evidence, revealing an association between serum ADMA levels and frailty status as assessed by the Fried metrics [51]. One potential mechanism that may underlie this result is the increased levels of inflammation and oxidative stress commonly seen in frail individuals. This may contribute to endothelial dysfunction by reducing the expression of dimethylarginine dimethylaminohydrolase (DDAH) [61], the enzyme responsible for ADMA metabolism. One of the effects of an increased serum ADMA concentration is a reduction in nitric oxide synthase activity and, consequently, nitrite levels. In this respect, a number of studies have demonstrated decreased nitrite levels in frail individuals (Table 4). In a cohort of 180 elderly Italian adults, Valdiglesias et al. [31] showed a significant increase in serum neopterin and C-reactive protein concentrations and a decrease in serum tryptophan and nitrite levels. Neopterin and tryptophan are two molecules with a metabolism that is closely linked with the mechanisms underlying subclinical chronic inflammation associated with aging [62]. However, of the four parameters mentioned, only the association of serum nitrite values with frailty was confirmed after multiple regression analysis. A similar study was performed by Mohamad et al. [23], in which decreased serum nitrite levels and increased neopterin and IFN-γ levels were found in frail individuals. IFN-γ is a cytokine that is intricately involved in neopterin biosynthesis, due to the transcriptional activation of the rate-limiting enzyme pteridin’s biosynthesis [63]. However, it is worth mentioning an important limitation of the latter study, namely, the lack of adjustment of the results for confounders, which limits the validity of this study.

### 3.7. Exploring Renal Endothelial Dysfunction in Frailty: Biomarkers of Renal Function

We examined the complex interactions between inflammation, cardiovascular health, and physical decline in our thorough examination of frailty. The complexity of this issue is further compounded by new research that clarifies the renal aspect of endothelial dysfunction and its consequences for frailty (Table 4). In this regard, in the Brain in Kidney Disease (BRINK) study [46], lower eGFR and higher UACR were related to decreased physical performance, with UACR appearing to be a significant factor even after adjusting for various risk factors. This finding differs from some previous studies, in which no link was found between eGFR and frailty after adjustment for risk factors [64], highlighting the nuanced relationship between chronic kidney disease (CKD) and physical function. However, while some studies have shown associations between eGFR and physical function, the significance of these associations is often diminished after adjusting for comorbidities and other factors prevalent in CKD [65]. In this context, this study revealed a robust association between albuminuria and reduced physical function, highlighting the importance of considering renal biomarkers in assessing the risk of frailty. However, the need for further longitudinal studies to elucidate the predictive value of albuminuria in tracking functional decline over time is underlined.

**Table 4:** Overview of the current studies supporting the association between biomolecular markers of endothelial dysfunction and frailty.

Biomolecular Markers for Assessment of Endothelial Dysfunction	Results	References
miRNA	Two interventional studies by Mone et al. show that empagliflozin improves cognitive impairment and physical decline by attenuating the mitochondrial calcium overload and reducing the production of reactive oxygen species in endothelial cells. Treatment also down-regulates the microRNAs associated with endothelial dysfunction, suggesting a potential link between frailty and endothelial dysfunction.	Mone et al. [29,66]
Inflammatory markers (e.g., Hcy, CRP, IL6, TNF-α)	Chronic inflammation is a key factor in frailty and cardiovascular pathology, with homocysteine and C-reactive protein playing key roles in influencing health. In this regard, elevated levels of Hcy and hsCRP have been linked to frailty. A study using artificial intelligence identifies immune biomarkers of aging and CXCL9’s role in cardiovascular pathology, highlighting the importance of inflammation in cardiovascular health.	Guillotin et al. [22], Álvarez-Sánchez et al. [37], Marcos-Pérez et al. [40], Sayed et al. [41]
Markers involved in NO signalling (e.g., ADMA, NO, neopterin)	Asymmetric dimethyl arginine (ADMA) is a potential biomarker of frailty in people without cardiovascular disease. Studies suggest increased inflammation and oxidative stress in frail individuals, potentially reducing ADMA metabolism and nitric oxide synthase activity. However, only serum nitrite levels have been found to be associated with frailty.	Mohamad et al. [21], Valdiglesias et al. [29], Alonso-Bouzón et al. [38]
Biomarkers of renal function (e.g., eGFR, UACR)	In a study conducted by Mello et al., the complex relationship between inflammation, cardiovascular health, and physical decline in frailty is explored, revealing a link between lower eGFR and increased UACR, and a robust association between albuminuria and reduced physical function.	Mello et al. [46]

### 3.8. Certainty of Evidence

Most of the studies discussed in our systematic review used observational designs, e.g., cross-sectional and cohort studies. These designs have a limited ability to establish causality because of potential bias and confounding, even though they offer insightful information about the relationships between exposures and outcomes. Because the studies are observational in nature, the degree of certainty regarding the evidence linking endothelial dysfunction and frailty is low.

A review of the included studies’ risk of bias raised a number of issues. The studies differed in terms of how they minimized selection bias; some used strict sampling techniques, while others had unclear participant selection procedures. Overall, selection bias was a moderate source of concern. Low measurement bias was also a result of the majority of studies using standardized techniques to evaluate endothelial dysfunction and frailty. To varying degrees across the studies, statistical adjustments and sensitivity analyses were used to try to address potential confounding. Nonetheless, in certain studies, ambiguity emerged as a result of there being inadequate explanations or strategies to address the confounding variables.

The majority of the included studies reported consistent findings regarding the relationship between endothelial dysfunction and frailty, despite differences in the study populations and methodologies. This consistency raises the level of confidence in the observed associations, improving the certainty of the evidence.

In line with this review’s research question, the included studies directly investigated the connection between endothelial dysfunction and frailty in clinical settings. As a result, the evidence is regarded as direct, confirming its certainty.

The studies differed in terms of the precision of their effect estimates; some with small sample sizes reported wide confidence intervals. Consequently, there is some concern regarding imprecision.

As the majority of the studies were observational, the GRADE approach was initially used to rate the certainty of the evidence for the association between endothelial dysfunction and frailty as low. On the basis of the directness of the evidence and the consistency of the findings, this rating might be raised to moderate. Overall confidence in the observed association may be affected by selection bias, measurement bias, confounding, and imprecision, all of which are causes for moderate concern.

## 4. Discussion and Conclusions

Our research points out an increasingly significant positive correlation between vascular endothelial dysfunction and frailty, while also highlighting common pathophysiological mechanisms that might represent targets for future anti-frailty drugs. According to the clinical research that we reviewed, this association is especially evident among older people without cardiovascular diseases.

**Figure 2:**
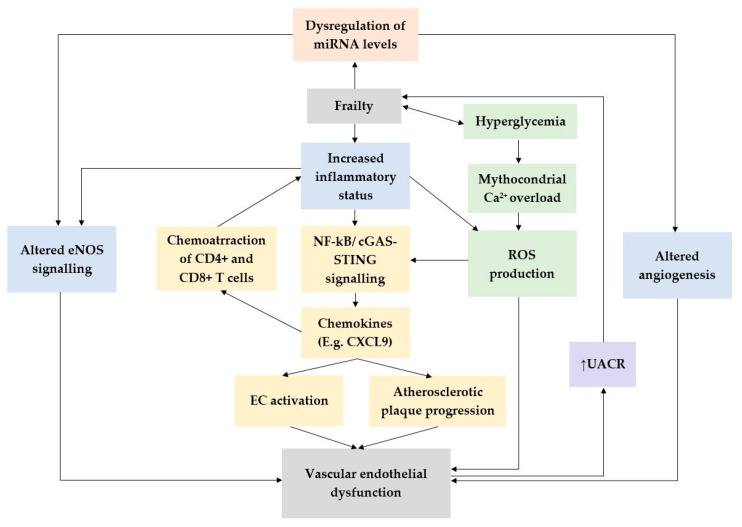
Possible associations between endothelial dysfunction and frailty. eNOS—endothelial nitric oxide synthase, ROS—reactive oxygen species, NF-kB—nuclear factor kappa-light-chain-enhancer of activated B cells, UACR—urine albumin–creatinine ratio.

Multisystem dysregulation is deeply linked to frailty syndrome, and a significant amount of evidence points to the involvement of multiple pathophysiological processes, including immune activation and chronic inflammation [67]. In this regard, there are a number of common underlying mechanisms, including chronic inflammation and oxidative stress, which are key links to frailty and endothelial dysfunction (Figure 2). Thus, the current literature indicates that chronic inflammation may serve as a central mechanism contributing to frailty through direct and indirect means [68]. Both clinical and biomedical studies have demonstrated elevated levels of molecular biomarkers, including interleukin-6 (IL-6), homocysteine, highly sensitive C-reactive protein (hs-CRP), neopterin, etc., associated with frailty, suggesting the critical role of chronic inflammation in the pathogenesis of frailty [68,69]. An important marker of endothelial dysfunction and also an emerging cardiovascular prognostic factor is the albumin–creatinine ratio, with both clinical and paraclinical studies showing the association of UACR↑ with a higher incidence of cardiovascular pathology and/or incident frailty [70,71].

One of the key actors in the pathogenesis of endothelial dysfunction is nitric oxide. The current evidence indicates the disruption of the NO signalling pathways and metabolism in frail older people. Moreover, this finding is corroborated by studies conducted in aged mouse models. For example, in aged animal models, treatment with the NO precursor sodium nitrite for eight weeks not only reduced low-grade inflammation but also attenuated the decline in motor function [47]. Following eight weeks of sodium nitrite treatment, the mice exhibited lower plasma levels of inflammatory markers. Additionally, their motor performance, particularly grip strength, showed an improvement compared to the control group [47]. These results in animal models emphasize the relationship between endothelial dysfunction and decreased NO availability and physical frailty or muscular weakness.

Aging is characterized by chronic low-grade inflammation, referred to as inflammaging, which contributes to endothelial dysfunction. Inflammatory processes activate signalling pathways such as NF-κB and cGAS-STING, promoting endothelial dysfunction and affecting vasorelaxation [72]. In this context, it is important to highlight the emerging role of the chemokine CXCL9, which is also known as interferon gamma-induced monokine (MIG). It is mainly secreted by activated macrophages and endothelial cells in response to inflammatory stimuli, and its role in endothelial dysfunction can be explained by a number of mechanisms in which it is involved. First, CXCL9 acts as a chemoattractant for T lymphocytes, particularly CD4+ and CD8+ T cells, which play crucial roles in the inflammatory response. At the same time, CXCL9 can activate endothelial cells to produce pro-inflammatory cytokines and adhesion molecules, further promoting inflammation and endothelial dysfunction. This may exacerbate the pro-inflammatory environment associated with aging and contribute to the progression of age-related cardiovascular disease [59]. Notably, the increased expression of CXCL9 has been observed in atherosclerotic lesions, where it contributes to inflammatory cell recruitment and the progression of plaque formation [73].

In this context, the emerging role of microRNAs as markers of endothelial dysfunction should not be overlooked. Numerous studies highlight the role of various miR subtypes (e.g., miR-21, miR-92, miR-126 etc.) and the mechanism of angiogenesis, immune activation, the induction of eNOS synthesis, etc. [13]. As the levels of these miRs increase, frailty is highlighted, which further supports the link between endothelial dysfunction and frailty. The administration of the SGLT2 inhibitor empagliflozin seems to play an important role in improving the levels of these miRNAs. It acts both by modulating the levels of miRNAs involved in endothelial dysfunction and by lowering glucose levels. In this regard, high glucose concentrations are associated with mitochondrial Ca^2+^ overload and significantly increased ROS production [29,66].

### Strengths and Limitations of This Review

Although the PRISMA statement, PRISMA checklist, and flowchart were followed in this systematic review, the review procedures that were used have some built-in limitations.

Firstly, even though we intended to create an all-encompassing search strategy, it is important to recognize the possibility that we overlooked pertinent studies. Language bias may have been introduced as a result of the language restrictions that limited the inclusion of studies published in languages other than English. Furthermore, because the search was restricted to electronic databases, it is possible that pertinent studies that were not indexed in these databases were missed.

Secondly, even though every attempt was made to reduce bias in the study selection and data extraction procedures, it is still possible that the inclusion or exclusion of studies and the data extraction process were influenced by subjective opinions. Even with multiple reviewers and predetermined criteria, bias might still be present to some extent.

Thirdly, even with the use of well-respected instruments such as the critical appraisal tools of the Joanna Briggs Institute, the evaluation of the quality of the included studies is intrinsically subjective. Divergent reviewers may assess the risk of bias differently, and conflicts may surface throughout the quality assessment procedure. Furthermore, the quality assessment may not have fully captured other possible sources of bias because it concentrated primarily on methodological issues.

This review may also be limited by the availability and quality of the included studies. The majority of studies were observational in nature, meaning that their ability to establish causality is limited. Furthermore, the quality of evidence for the association between frailty and endothelial dysfunction was generally rated as low to moderate, reflecting the limitations of the available literature.

## Figures and Tables

**Table 1:** Key search terms and strategies.

Database		Search Term/Phrase	Limit
Scopus, ScienceDirect, Medline	Concept 1 AND	“vascular endothelium” OR “endothelial dysfunction” OR “endothelial function” OR “nitric oxide” OR “vascular elasticity” OR “subclinical atherosclerosis” OR “subclinical cardiovascular disease” OR “arterial stiffness”	Articles in English, Time period: January 2014–January 2024, original peer-reviewed articles
	Concept 2	“frailty” OR “frail”	

**Table 2:** Selection criteria for articles included in the systematic review.

Parameter Used	Inclusion Criteria	Exclusion Criteria
Language	English	Any language other than English
Access	Free access to the full text	Access to the full text, financially conditional
Species	Human subjects	Non-human subjects
Type of study	Observational, cohort, case–control, or multicentre randomized trials	Meta-analysis, systematic review, case studies, editorials
Period of publication	Studies published in the last 10 years (2014–2024)	Studies published before 2014
Duplicate	No	Yes

**Table 3:** Overview of the current studies supporting the association between physiological markers of endothelial dysfunction and frailty.

Non-Invasive Markers for Assessment of Endothelial Dysfunction	Results	References
FMD	Flow-mediated vasodilation (FMD) is a marker that measures the endothelium’s ability to respond to shear stress. Studies have shown that frail patients have a lower frequency of FMD ≥ 10% compared to non-frail patients, especially in those with chronic kidney disease stages 3–5.	Mansur et al. [17], Park et al. [30]
PWV	Researchers found that pre-frail and frail individuals had significantly higher values for pulse wave velocity (PWV), a widely used measure of arterial stiffness. However, a study by Kannegieter et al. [33] on elderly patients failed to show a correlation between frailty and aortic stiffness.	Macêdo et al. [16], Jiang et al. [18,19], Papaioannou et al. [23], Yamanashi et al. [24], Park et al. [30], Kannegieter et al. [33], McKechnie et al. [34,35], Lim et al. [36], Álvarez-Bustos et al. [39], Orkaby et al. [42], Nadruz et al. [43]
RHI	Reactive hyperaemia peripheral arterial tonometry (RH-PAT) measures flow-induced arterial dilation using the reactive hyperaemia index (RHI). A study found an association between endothelial dysfunction and grip force in 236 elderly women, but this association has not been confirmed in other studies.	Yoo et al. [32], Lim et al. [36]
CIMT	Carotid intima–media thickness (CIMT), a marker of arterial stiffness, has been found to be positively correlated with frailty. Furthermore, in a longitudinal descriptive study by Veronese et al., frail patients had more moderate or severe carotid plaques and a higher coronary calcium score, leading to a higher cumulative incidence of cardiovascular disease.	Xue et al. [20], Yamanashi et al. [24], Veronese et al. [26], Park et al. [31], McKechnie et al. [34,35], Lim et al. [36]
ABPI	The ankle–brachial index (ABPI) is a key parameter in patients with suspected peripheral arterial disease and atherosclerosis. Studies have shown an association between abnormal values of PWV and ABPI and frailty.	Xue et al. [20], McKechnie et al. [34,35], Nadruz et al. [43]
CAVI	CAVI, an index of global arterial stiffness, has been linked to sedentary behaviour in Japanese patients with frailty. Studies have also shown a negative association between arterial stiffness and grip strength in non-hypertensive patients. In a study by Xue et al., CAVI was identified as an independent risk factor for frailty, highlighting the importance of understanding and managing arterial stiffness.	Xue et al. [20], Yamanashi et al. [24], Shiraishi et al. [25]
cxPA	Blood pressure complexity (cxPA) is a measure of the pathophysiological mechanisms influencing cardiovascular regulation in the elderly. Studies show that lower systolic or diastolic BP complexity increases the risk of pre-frailty or frailty, and an inverse correlation exists between arterial stiffness and cxPA, suggesting cxPA acts as a mediator between these factors.	Jiang et al. [20,21]

## Data Availability

The original contributions presented in the study are included in the article/Supplementary Materials; further inquiries can be directed to the corresponding authors.

## Supplementary Materials

The following supporting information can be downloaded at: https://www.mdpi.com/article/10.3390/jcm13092686/s1, Supplementary Chapter S1 (Sup 1)—Systematic review methods; Supplementary Chapter S2 (Sup 2)—Reasons for article exclusions at the full-text review stage; Table S1—PRISMA 2009 Checklist; Table S2—Recent published literature confirming association between endothelial dysfunction/subclinical atherosclerosis and frailty; Table S3—Critical Appraisal of the included studies.
