# Supplementary material for: Endothelial Dysfunction as a Key Link between Cardiovascular Disease and Frailty: A Systematic Review
Source: J Clin Med. 2024 May 2;13(9):2686. doi: 10.3390/jcm13092686 (PMC11084631; doi:10.3390/jcm13092686)
Supplement: Supplementary file 1 [file jcm-13-02686-s001.zip › jcm-2959565-supplementary.pdf]

Systematic Review

Endothelial Dysfunction as a Key Link between Cardiovascular Disease and Frailty

SUPPLEMENTARY MATERIALS

- Supplementary Chapter S1 (Sup 1)—Systematic review methods
- Supplementary Chapter S2 (Sup 2)—Reasons for article exclusions at the full-text review stage
- Table S1—PRISMA 2009 Checklist
- Table S2—Recent published literature confirming association between endothelial dysfunction/subclinical atherosclerosis and frailty
- Table S3—Critical Appraisal of the included studies
- References

Supplementary Chapter S1

Methods of systematic review

Preferred Reporting topics of Systematic Reviews and Meta-analysis (PRISMA) Guidelines [1] (Figure 1) were followed when conducting this study, and the PRISMA 2009 checklist was utilized to organize and document the review topics (Supplementary Table 1). We have to register our study protocol within the International Prospective Register of Systematic Reviews (PROSPERO).

S1.1 Search Strategy

We conducted a comprehensive online search of medical databases, including Scopus, ScienceDirect, and Medline, between January 2014 and January 2024. This specific time period was selected to encompass all of the relevant and recently published literature. Additionally, our goal in using this approach was to incorporate all of the most recent materials published since the last review on this topic[2] to date. A summary of the key search terms and strategies is provided in Table 1. The search strategy was developed by the lead author in conjunction with the associates.

S2.2 Selection criteria

This systematic review includes all original clinical research studies that assessed the relationship, in clinical settings, between nitric oxide signalling and vascular endothelial dysfunction and physical frailty. The articles had to provide a quantitative assessment of the correlation in a clinical environment between vascular endothelial dysfunction or nitric oxide signalling and physical frailty. Only original, peer-reviewed clinical research studies written in English were included. Excluded from consideration were narrative reviews, editorials, conference pieces, theses, methodological articles, systematic literature reviews and meta-analyses, and works reporting animal models.

S2.3 Literature retrieval and selection

A three-phase search plan was applied. After performing a preliminary, restricted search of Medline and Google Scholar, text terms found in the titles and abstracts as well as the index terms used to characterize the articles that were found were analysed. All included databases were subjected to a second search that made use of all identified key phrases and index keywords. Lastly, a manual search was conducted for more pertinent studies in the reference lists of the selected relevant papers. Duplicates were eliminated after the search. The first round of title and abstract screening was performed individually by all of the authors: H.C., E.B., R.I.N., and D.A.I. Additionally, a manual search of

the articles' reference list was performed using the specified inclusion and exclusion criteria. In order to choose the final papers for inclusion in the review, the three authors H.C., E.B., and R.I.N. independently approved each full text of every article that satisfied the inclusion criteria once it had been obtained and reviewed separately. To find more pertinent research, the reference lists of the highlighted papers were also searched. The writers worked through any differences through dialogue. The Transparent Reporting of Non-randomised Designs (TREND) declaration[3] contained principles for discussing non-randomised designs.

S2.4 Data extraction and synthesis methods

We employed rigorous criteria to determine the eligibility of studies for inclusion in our synthesis. These criteria were established a priori and were based on the objectives of our review, focusing on original clinical research articles published in English between January 2014 and January 2024. Studies had to report quantitative assessments of the relationship between frailty and vascular endothelial dysfunction in a clinical setting.

After selecting the eligible studies, we carefully prepared the data for presentation and synthesis. This included dealing with missing summary statistics, such as extracting the missing data from related articles when the study design was described in detail in other articles, or contacting the study authors for clarification. In addition, data conversions were carried out where necessary to ensure the uniformity of the studies and to facilitate comparison.

We utilized various methods to tabulate and visually display the results of individual studies and syntheses. The primary author HC extracted data using tabular summaries and discussed it with the research team. Data included first author, year of publication, reference, study design, participant characteristics and age (if available), sample size, sample stratification (if available), vascular endothelial dysfunction and physical frailty outcome measures, results, and author's conclusion. The authors H.C. and E.B. worked together to address any differences before completing the literature overview table.

The synthesis of results was guided by the objectives of our review and the nature of the included studies. Given the heterogeneity of study designs and outcome measures, a narrative synthesis approach was primarily employed to integrate findings across studies. This involved identifying common themes and patterns, comparing and contrasting study results, and providing a comprehensive overview of the evidence base.

S2.5 Quality appraisal of the selected studies for the review

H.C. and E.B. independently evaluated the risk of bias within and among the chosen studies using the Joanna Briggs Institute Critical Appraisal tools. We chose to utilize two of the JBI Critical assessment instruments in order to accurately assess the quality of our studies, taking into account the study design, because the studies included in the current review varied widely with regard to their design. As a result, the JBI critical appraisal tool for use in analytical cross-sectional studies (JBI tool) and the JBI critical appraisal tool for cohort studies were chosen as the instruments for quality appraisal[4]. The Joanna Briggs Institute (2017) states that this process allowed for higher methodological rigor and analysed potential bias and risks to validity (https://reviewersmanual.joannabriggs.org/). The two reviewers were trained to use the appraisal instrument before this process began. Table 2 provides the details of the quality appraisal of the selected studies included in this review.

In a nutshell, the eight questions that made up the JBI tool for cross-sectional research are as follows: (Q1) Were the criteria for inclusion in the sample clearly defined? (Q2) Were the study subjects and the setting described in detail? (Q3) Was the exposure measured in a valid and reliable way? (Q4) Were objective, standard criteria used for measurement of the condition? (Q5) Were confounding factors identified? (Q6) Were strategies to deal with confounding factors stated? (Q7) Were the outcomes measured in a valid and reliable way? (Q8) Was appropriate statistical analysis used? Each criterion could be answered in four ways: “Yes”, “No”, “Unclear”, and “Not applicable”.

Conversely, the following questions are part of the JBI critical appraisal tool for cohort studies: (Q1). Were the two groups similar and recruited from the same population? (Q2). Were the exposures measured similarly to assign people to both exposed and unexposed groups? (Q3). Was the exposure measured in a valid and reliable way? (Q4).

Were confounding factors identified? (Q5). Were strategies to deal with confounding factors stated? (Q6). Were the groups/participants free of the outcome at the start of the study (or at the moment of exposure)? (Q7). Were the outcomes measured in a valid and reliable way? (Q8). Was the follow-up time reported and sufficient to be long enough for outcomes to occur? (Q9). Was follow-up complete, and if not, were the reasons for loss to follow-up described and explored? (Q10). Were strategies to address incomplete follow-up utilized? (Q11). Was appropriate statistical analysis used?

S2.6. Reporting bias assessment

In the absence of a meta-analysis, identifying and addressing potential sources of bias within individual studies was the primary focus of the reporting bias assessment. We used a number of techniques to reduce the possibility of bias resulting from missing data. First, by looking through a variety of electronic databases and incorporating research that were published in English between January 2014 and January 2024, our thorough search approach sought to reduce publication bias. In addition, we evaluated the included studies critically for bias in reporting, including selective outcome reporting. We specifically looked for differences between the results reported in the results sections and the methods sections of the included studies in order to find any discrepancies. Furthermore, we assessed the risk of bias using the Joanna Briggs Institute's critical appraisal tools to evaluate study quality and risk of bias across various domains as described in the previous section.

S2.7. Certainty assessment

As advised by the PRISMA guidelines, we used a qualitative method based on the GRADE principles[5] to evaluate the degree of certainty in the evidence for each outcome. Due to the fact that for the majority of the articles included in this review the common outcome was endothelial dysfunction, the focus of the certainty assessment was on this outcome only. This required assessing the overall quality of evidence from all of the included studies, taking into account variables like study design, bias risk, consistency of findings, and estimate precision.

Our review included observational studies as well as potentially other study designs, so we classified the certainty of evidence for endothelial dysfunction as the main common outcome as either high, moderate, low, or very low. The methodological rigor, consistency, and strength of the correlation found between endothelial dysfunction and frailty in the body of evidence all influenced this qualitative assessment.

Supplementary Chapter S2—Reasons for article exclusions at the full-text review stage

Reasons for article exclusions at the full-text review stage

The following factors led to the exclusion of 55 of the 84 papers that were chosen for the full-text review:

- Studies conducted on non-human subjects/cell cultures (n=11);
- Articles published in a language other than English (n=6);
- Articles reporting mechanisms other than endothelial dysfunction or subclinical cardiovascular disease and/or not correlating the markers of endothelial dysfunction with frailty or frailty-associated mechanisms (n = 38).

Table S1. PRISMA 2020 Checklist

Section and Topic	Item #	Checklist item	Location where item is reported
**TITLE**			
Title	1	Identify the report as a systematic review.	Page 1
**ABSTRACT**			
Abstract	2	See the PRISMA 2020 for Abstracts checklist.	Page 1
**INTRODUCTION**			
Rationale	3	Describe the rationale for the review in the context of existing knowledge.	Pages 1 and 2
Objectives	4	Provide an explicit statement of the objective(s) or question(s) the review addresses.	Page 2
**METHODS**			
Eligibility criteria	5	Specify the inclusion and exclusion criteria for the review and how studies were grouped for the syntheses.	Pages 2 and 3
Information sources	6	Specify all databases, registers, websites, organisations, reference lists, and other sources searched or consulted to identify studies. Specify the date when each source was last searched or consulted.	Page 2 and Sup 1
Search strategy	7	Present the full search strategies for all databases, registers, and websites, including any filters and limits used.	Table 2 and Sup 1
Selection process	8	Specify the methods used to decide whether a study met the inclusion criteria of the review, including how many reviewers screened each record and each report retrieved, whether they worked independently, and if applicable, details of automation tools used in the process.	Table 3 and Sup 1
Data collection process	9	Specify the methods used to collect data from reports, including how many reviewers collected data from each report, whether they worked independently, any processes for obtaining or confirming data from study investigators, and if applicable, details of automation tools used in the process.	Sup 1

Data items	10a	List and define all outcomes for which data were sought. Specify whether all results that were compatible with each outcome domain in each study were sought (e.g., for all measures, time points, analyses), and if not, the methods used to decide which results to collect.	Table 4
	10b	List and define all other variables for which data were sought (e.g., participant and intervention characteristics, funding sources). Describe any assumptions made about any missing or unclear information.	Sup 1 and Table 4
Study risk of bias assessment	11	Specify the methods used to assess risk of bias in the included studies, including details of the tool(s) used, how many reviewers assessed each study and whether they worked independently, and if applicable, details of automation tools used in the process.	Page 4 and Sup 1
Effect measures	12	Specify for each outcome the effect measure(s) (e.g., risk ratio, mean difference) used in the synthesis or presentation of results.	Table 4
Synthesis methods	13a	Describe the processes used to decide which studies were eligible for each synthesis (e.g., tabulating the study intervention characteristics and comparing against the planned groups for each synthesis (item #5)).	Page 3 and Sup 1
	13b	Describe any methods required to prepare the data for presentation or synthesis, such as handling of missing summary statistics, or data conversions.	Sup 1
	13c	Describe any methods used to tabulate or visually display results of individual studies and syntheses.	Sup 1
	13d	Describe any methods used to synthesise results and provide a rationale for the choice(s). If meta-analysis was performed, describe the model(s), method(s) to identify the presence and extent of statistical heterogeneity, and software package(s) used.	Page 4 and Sup 1
	13e	Describe any methods used to explore possible causes of heterogeneity among study results (e.g., subgroup analysis, meta-regression).	N/A
	13f	Describe any sensitivity analyses conducted to assess the robustness of the synthesised results.	N/A
Reporting bias assessment	14	Describe any methods used to assess risk of bias due to missing results in a synthesis (arising from reporting biases).	Sup 1
Certainty assessment	15	Describe any methods used to assess certainty (or confidence) in the body of evidence for an outcome.	Page 4 and Sup 1
**RESULTS**			
Study selection	16a	Describe the results of the search and selection process, from the number of records identified in the search to the number of studies included in the review, ideally using a flow diagram.	Page 5

	16b	Cite studies that might appear to meet the inclusion criteria, but which were excluded, and explain why they were excluded.	Page 5 and Sup 2
Study characteristics	17	Cite each included study and present its characteristics.	Page 5
Risk of bias in studies	18	Present assessments of risk of bias for each included study.	Table 5
Results of individual studies	19	For all outcomes, present, for each study: (a) summary statistics for each group (where appropriate) and (b) an effect estimate and its precision (e.g., confidence/credible interval), ideally using structured tables or plots.	Pages 6 to 10
Results of syntheses	20a	For each synthesis, briefly summarise the characteristics and risk of bias among contributing studies.	Tables 4 and 5
	20b	Present results of all statistical syntheses conducted. If meta-analysis was done, present for each the summary estimate and its precision (e.g., confidence/credible interval) and measures of statistical heterogeneity. If comparing groups, describe the direction of the effect.	N/A
	20c	Present results of all investigations of possible causes of heterogeneity among study results.	N/A
	20d	Present results of all sensitivity analyses conducted to assess the robustness of the synthesised results.	N/A
Reporting biases	21	Present assessments of risk of bias due to missing results (arising from reporting biases) for each synthesis assessed.	Table 5
Certainty of evidence	22	Present assessments of certainty (or confidence) in the body of evidence for each outcome assessed.	Page 11
**DISCUSSION**			
Discussion	23a	Provide a general interpretation of the results in the context of other evidence.	Pages 11 and 12
	23b	Discuss any limitations of the evidence included in the review.	Pages 11 and 12
	23c	Discuss any limitations of the review processes used.	Pages 13 and 14

	23d	Discuss implications of the results for practice, policy, and future research.	Pages 13 and 14
**OTHER INFORMATION**			
Registration and protocol	24a	Provide registration information for the review, including register name and registration number, or state that the review was not registered.	Sup 1
	24b	Indicate where the review protocol can be accessed, or state that a protocol was not prepared.	N/A
	24c	Describe and explain any amendments to information provided at registration or in the protocol.	N/A
Support	25	Describe sources of financial or non-financial support for the review, and the role of the funders or sponsors in the review.	Page 14
Competing interests	26	Declare any competing interests of review authors.	Page 14
Availability of data, code, and other materials	27	Report which of the following are publicly available and where they can be found: template data collection forms; data extracted from included studies; data used for all analyses; analytic code; and any other materials used in the review.	Page 14

Section and Topic	Item #	Abstract checklist item	Reported (Yes/No)
**TITLE**			
Title	1	Identify the report as a systematic review.	Yes
**BACKGROUND**			
Objectives	2	Provide an explicit statement of the main objective(s) or question(s) the review addresses.	Yes
**METHODS**			
Eligibility criteria	3	Specify the inclusion and exclusion criteria for the review.	Yes
Information sources	4	Specify the information sources (e.g., databases, registers) used to identify studies and the date when each was last searched.	Yes
Risk of bias	5	Specify the methods used to assess risk of bias in the included studies.	Yes
Synthesis of results	6	Specify the methods used to present and synthesise results.	Yes
**RESULTS**			
Included studies	7	Give the total number of included studies and participants and summarise the relevant characteristics of studies.	Yes

Synthesis of results	8	Present results for main outcomes, preferably indicating the number of included studies and participants for each. If meta-analysis was performed, report the summary estimate and confidence/credible interval. If comparing groups, indicate the direction of the effect (i.e., which group is favoured).	Yes
**DISCUSSION**			
Limitations of evidence	9	Provide a brief summary of the limitations of the evidence included in the review (e.g., study risk of bias, inconsistency, and imprecision).	Yes
Interpretation	10	Provide a general interpretation of the results and important implications.	Yes
**OTHER**			
Funding	11	Specify the primary source of funding for the review.	Yes
Registration	12	Provide the register name and registration number.	Yes

Table S2: Recent published literature supporting the association between endothelial dysfunction/subclinical atherosclerosis and frailty.

**Clinical outcomes of endothelial dysfunction**				
**Author/ Year**	**Study design and setting**	**Assessment of endothelial dysfunction**	**Results and outcome**	**Conclusions**
Mansur et al. 2015 [6]	Descriptive cross-sectional study, initial cohort of 61 patients prediagnosed with BCR stages 3-5 at the IMEPEN Foundation Patient Clinic of the Federal University of Juiz de Fora, Brazil were enrolled in this study. Subsequently, 57 of them were re-evaluated after 12 months.<br>• Age: 60 ± 11.5 years<br>• Mean eGFR: 23 (16.0-39.0) mL/min/1.73 m2<br>• Prevalence of frailty: 42.6%, of whom 54% were elderly (defined in this study as people aged over 60)	Flow-mediated vasodilation (FMD)	Within the frail group there were 9 patients with FMD ≥10% (34.6%), whereas the non-frail group had 21 subjects with FMD ≥10% (60%).<br>After adjusting for confounding variables, frailty was found to be associated with gender (OR = 11.32; 95% CI = 2.30-55.67), older age (OR = 4.07; 95% CI = 1.0216.20), and obesity (OR = 6.63; 95% CI = 0.82-11.44).<br>Frailty was also associated with endothelial dysfunction (OR = 3.86; 95% CI = 1.00-14.88).<br>Reassessment of patients at 12-month follow-up showed that frailty accounted for a risk ratio of 2.5 (95% CI = 1.04-6.10), thus a higher risk of adverse health outcomes.	In this study, an increased prevalence of frailty was documented in a sample of Brazilian predialysis patients. We found that frailty is associated with gender (female), older age, obesity, and endothelial dysfunction.
Park et al. 2022[7]	Cross-sectional descriptive study, cohort of 92 older adults (> 65 years).<br>Non-frail: 30; Pre-frail: 43; Frail: 19	baPWV and FMD	PWV was significantly higher in both the pre-frail and frail groups than in the non-frail group (non-frail: 1615.7 ± 209.9 cm/s vs. pre-frail: 1815.2 ± 265.0 cm/s vs. frail: 1829.9 ± 256.0 cm/s, respectively, p = 0.003).<br>FMD was significantly lower in both the pre-frail and frail groups than in the non-frail group (non-frail: 5.1 ± 2.1% vs. pre-frail: 3.4 ± 1.3% vs. frail: 3.1 ± 1.2% cm/s, respectively, p = 0.001).<br>Multiple logistic regression analyses indicated that the pre-frail and frail groups were associated with arterial stiffness (OR, 2.92; 95% CI, 1.01-8.42; OR, 3.56; 95% CI, 0.85-14.91) and endothelial dysfunction (OR, 2.17; 95% CI, 0.41-3.09; OR, 2.27; 95% CI, 0.31-6.97).	Pre-frailty and frailty are associated with impaired vascular function in community-dwelling older adults, even when adjusting for confounders.

Álvarez-Bustos et al. 2023[8]	Longitudinal descriptive study, cohort of 978 participants with a mean age of 74.5 years +-5.6 years belonging to the Toledo Healthy Aging Study cohort. The population consisted of 700 non-frail individuals, 257 pre-frail individuals, and 21 frail individuals at the time of the initial assessment.	cfPWV	Different PWV cut-off points were identified for each outcome. PWV >11.5 m/s was cross-sectionally associated with frailty and disability, controlling for possible confounders (OR 95% CI 1.69 (1.45-1.97), p<0.001). The pulse wave velocity (PWV) cut-off for incident frailty was identified as 10 m/s. PWV exceeding 10 m/s was significantly associated with incident frailty in individuals initially classified as robust or non-frail (OR 95% CI 1.36 91.10-1.68, p<0.005). In contrast, in participants with PWV ≤ 10 m/s, age rather than PWV was a significant predictor of frailty development.	Arterial stiffness predicts frailty, disability, and mortality in older people, with distinct cut-offs indicating different levels of severity for each outcome. These findings highlight the importance of assessing arterial stiffness in older populations to predict functional decline and mortality.
Nadruz et al. 2016[9]	Cross-sectional descriptive study. The ARIC study, which began in 1987-1989, involved 15,792 participants aged 45-64 from four US communities. After evaluating 6,538 surviving participants from Visit 5 (2011-2013), the analysis included 3,991 individuals. The mean age was 75.6 ± 5.0 years, with 59.1% being female, 23.4% black, and 5.3% frail.	cfPWV, ABPI	Frailty was associated with worse vascular function, reflected by a higher prevalence of both ABPI (p=0.030) and abnormal PWV (0.012). In this sense, frailty was associated with the described vascular abnormalities (OR = 1.44; 95% CI = 1.06 - 1.95).	In the present study, vascular dysfunction, defined as abnormal PWV or ABPI, was independently related to frailty in analyses adjusted for demographic factors and abnormalities in other systems.
Kannegieter et al. 2016[10]	Cross-sectional descriptive study; cohort of 117 patients aged 60 years and older (median age 79 years) who visited the Erasmus MC geriatric clinic between April 2015 and August 2015. Frailty was assessed using the Fried Frailty Index. Thus, 27 were non-frail, 61 pre-frail, and 29 frail.	aPWV	Frail participants had significantly longer completion times for the Timed Up and Go (TUG) (p<0.001) and 5-meter walk (p<0.001) tests, along with lower prehension strength (p=0.001) compared to non-frail counterparts. However, there was no significant association between frailty and aortic stiffness (p=0.778).	The current study highlights the validity of the TUG, the 6-meter walk test and the HGS as screening tests for frailty. However, the current study could not reveal a relationship between aortic stiffness and frailty.
Macêdo et al. 2022[11]	Cross-sectional descriptive study involving 117 patients aged 60 and older. These visited the Erasmus MC geriatric clinic between April 2015 and August 2015. The study assessed frailty using the Fried Frailty Index. The study found that 27 patients were non-frail, 61 pre-frail, and 29 frail. The study also found that 61.8% of the patients were pre-frail, and 38.3 were non-frail (reddish). Both studies were conducted at Onofre Lopes University Hospital and the Physical Education Department of the Federal University of Rio Grande do Norte.	aPWV	Pre-frail older adults had higher arterial stiffness compared to robust individuals, with aPWV being significantly higher in the pre-frail group (β = 0.19 m/s; p = 0.007).	The pre-frail phenotype has been associated with increased arterial stiffness in adults aged 60-80 years. This suggests that pre-frail older adults may have an increased risk of cardiovascular disease.

Jiang et al. 2023[12]	Cross-sectional descriptive study; cohort of 442 participants with a mean age of 71.4 ± 8.1 years, 235 of whom were women, underwent assessments for frailty syndromes and various multisystemic conditions.<br>Frailty was assessed using the Fried Frailty Index. Thus, of the participants, 11.3% were frail, 48.0% pre-frail, and 40.7% non-frail or frail.	baPWV, BP complexity	This study found a direct link between poor vascular function and increased risk of sluggishness, weakness, and exhaustion. Lower SBP or DBP complexity was linked to higher odds of being pre-frail or frail ($p<0.05$). Those with higher baPWV were also more likely to be pre-frail ($p=0.018$) or frail ($p=0.032$).	This pilot study provides new insight into the associations between multisystemic conditions and frailty in older adults. Findings suggest that vascular dysfunction may contribute to physical frailty.
Jiang et al. 2022[13]	Cross-sectional descriptive study, conducted between January and October 2021, involving 350 older adults who underwent assessments for frailty, arterial stiffness, and beat-to-beat blood pressure. Blood pressure complexity was quantified using multiscale entropy analysis.<br>Frailty was assessed using the Fried Frailty Index. Thus, of the participants, 38 were frail, 170 were pre-frail, and 142 were non-frail or red.	baPWV, BP complexity	Compared to non-frail individuals, the pre-frail and frail groups had significantly lower systolic (SBP, $p<0.001$) and diastolic (DBP, $p<0.001$) beat-to-beat blood pressure complexity and higher arterial stiffness ($p<0.001$). Arterial stiffness was inversely associated with BP complexity. Mediation analyses showed that SBP and DBP beat complexity partially mediated the relationship between arterial stiffness and frailty, explaining approximately 47% of the total effect on frailty (mediated proportion: SBP: 50%, DBP: 47%).	This study highlights the association between BP complexity and frailty in older adults. Moreover, BP complexity was found to mediate the link between arterial stiffness and frailty, suggesting its potential utility as a marker for characterizing key physiological functions in older adults.
Yamanashi et al. 2018[14]	Cross-sectional descriptive study.<br>Participants included individuals aged ≥40 years enrolled in the third follow-up examination of the Andhra Pradesh Children and Parents Study, India (1506 participants), and in the initial evaluations of the Nagasaki Islands Study, Japan (3166 participants). These were investigated in establishing an association between hand-grip strength (HGS) and markers of subclinical atherosclerosis.	baPWV, CIMT, CAVI	CIMT showed a negative association with HGS in non-hypertensive Indian men (B coefficient = -5.38, $P = 0.036$). Arterial stiffness was also associated with HGS in non-hypertensive Indian men (B = -0.97, $P = 0.001$), but not in hypertensive Indian men. Similarly, significant associations between arterial stiffness and HGS were observed in non-hypertensive women in both India and Japan (B = -0.44, $P = 0.020$, B = -0.63, $P = 0.016$, respectively), but not in hypertensive women.	This study highlights a negative relationship between preclinical atherosclerosis and HGS. At the same time, HGS being an important indicator of frailty, this study suggests an association between frailty and subclinical atherosclerosis.
Orkaby et al. 2019[15]	Cross-sectional descriptive study, cohort of 2171 participants from the Framingham Heart Study Offspring and Omni cohorts aged 60 years and older, investigated between 2005 and 2008.<br>Frailty was primarily assessed using the Fried physical phenotype definition, which classifies individuals as non-frail, pre-frail, or frail. Thus, 45% of individuals were pre-frail and 7% of individuals were frail.	cfPWV	Adjusted analysis revealed a significant association between frailty level and cfPWV, with a higher cfPWV observed in pre-frail and frail individuals compared to non-frail counterparts ($p=0.0002$). This association persisted even after adjusting for various confounders (e.g., diabetes and coronary heart disease, $p=0.06$).	Findings suggest that pre-frailty and frailty are related to increased arterial stiffness in older adults. This association highlights the potential role of arterial stiffness in explaining the relationship between frailty and cardiovascular disease.

Papaioannou et al. 2014[16]	Longitudinal descriptive study, initial cohort of 279 older adults (mean age 85.5+-7.0 years) who were followed up for a mean of 12.8=- 6.3 months.<br>TC was calculated using the formula TC=k×PWV-2, with the coefficient k adjusted for body mass index (BMI). Survivors (n=185) and non-survivors (n=94) were compared in terms of PWV and TC.	TC (total arterial compliance), cfPWV	Non-survivors showed similar PWV compared to survivors (14.9±3.8 m/s vs. 14.2±3.6 m/s, respectively; p=0.139), while TC was significantly lower in non-survivors than in survivors (0.198±0.128 ml/mmHg vs. 0.221±0.1 ml/mmHg; p=0.018). CT emerged as a significant predictor of mortality (p=0.022, odds ratio=0.326), even after adjusting for sex, mean pressure, and heart rate, whereas PWV was not predictive (p=0.202). Age was independently associated with CT (p=0.016), but not with PWV.	In the elderly, CT estimated using a new method has demonstrated the ability to predict all-cause mortality. This suggests that CT may serve as a more sensitive arterial biomarker for CV risk assessment compared to PWV, and also highlights its role in assessing the association between vascular dysfunction and age, i.e., frailty.
McKechnie et al. 2021[17]	Longitudinal descriptive study, initial cohort of 7735 British Regional Heart Study men aged 40-59 years at enrolment. Initial screening took place in 1978-1990. A 30-year review took place in 2010-2012, in which all 3137 surviving men were invited to participate. Of these, 1057 men were assessed over a 3-year period and included in the current study. Subsequently, follow-up data were available for 865 patients.<br>Men with a previous diagnosis of AMI, stroke, and/or HF at baseline and those who were frail at baseline were excluded. Men missing all four subclinical CVS markers (DC, ABPI, cfPWV, and CIMT) were excluded.	cfPWV, CIMT, DC, ABPI	78 adults became frail.<br>In multivariate analyses, a higher CIMT value was associated with higher odds of incident frailty compared to those in the first tertile of CIMT (third tertile OR 2.70, 95% CI 1.40-5.20, p=0.003).<br>This was only slightly attenuated when further adjusting for confounders (third 3 OR 2.61, 95% CI 1.30-5.23, p=0.007).<br>There were weaker, non-statistically significant associations between higher cfPWV and higher odds of incident frailty (2nd tertile OR 1.86, 95% CI 0.92-3.77, p=0.08, 3rd tertile OR 1.76, 95% CI 0.87-3.57, p=0.12, between-group trend p=0.15).<br>There were no clear associations between CD or ABPI and incident frailty.	The current study extends existing cross-sectional associations previously reported by demonstrating a longitudinal association between subclinical CVD and incident frailty. Given additional evidence suggesting that clinically apparent CVD is a risk factor for frailty and vice versa, it seems plausible that CVD and frailty share common causal mechanisms.
Lim et al. 2021[18]	Cross-sectional descriptive study, cohort of 336 participants.<br>Median age (interquartile range (IQR)) of 62 (59-67) years; they were mostly male (55.1%) and Chinese (82.1%).	CIMT, aortic stiffness (cfPWV, aAIx, aPP) , carotid stiffness (DC), RHI	The study found significant inverse associations between cIMT (p<0.001), carotid stiffness (p<0.05), aortic stiffness (p<0.05), and gait speed, with no significant association with muscle mass and function. Despite adjustments, cIMT remained inversely associated with walking speed. Age-related interactions were found in aortic stiffness and gait speed associations.	The results support a link between systemic vascular health and skeletal muscle mass and function in middle-aged and older Asian adults. This association may be best reflected by cIMT, given its independent association with muscle strength and function, the main determinants of sarcopenia, in older adults.
McKechnie et al. 2022[19]	Cross-sectional descriptive study, cohort of 1399 patients, selected from the original cohort of 3137 patients of the British Regional Heart Study [20] alive at the time of examination.<br>• Age: 71-92 years.	CIMT, DC, ABPI, and cfPWV in both those with and without CVD.<br>vWF in both groups.	The study found that certain factors, including vWf, CIMT, ABPI < 0.9, and cfPWV, were positively associated (p<0.05) with frailty in the group without CVD, while DC was negatively associated with frailty. However, in the no-	In this cohort of older British men, biomarkers of endothelial dysfunction were strongly associated with prefrailty and frailty in men without clinically manifest cardiovascular disease, as were some

	• Of the 1399 patients: 1096 had no CVD and 303 had CVD.<br>• 82/303 (27%) and 168/303 (55%) of men with CVD were frail and pre-frail, respectively.<br>• 152/1096 (14%) and 603/1096 (55%) of men without CVD were frail and pre-frail, respectively.		CVD group, vWF showed a statistically positive association with pre-frailty and frailty, while cfPWV was positively associated with pre-frailty but weakly associated with frailty. ABPI < 0.9 and frailty showed a positive but insignificant association, while DC showed a strong negative association with prefrailty and frailty. In a subsequent model, only DC remained statistically significantly associated with frailty. In men with prevalent cardiovascular disease, log vWF showed no clear association with frailty or pre-frailty.	imaging markers of subclinical vascular dysfunction (carotid distensibility and cfPWV). In contrast, among men with cardiovascular disease, biomarkers of endothelial dysfunction were not associated with frailty. The results also suggest that carotid stiffness is independently associated with frailty status in men without clinically evident CVD.
Veronese et al. 2017[21]	Longitudinal descriptive study, initial cohort of 5764 subjects aged > 65 years from the Reykyavik study.[21] They were followed up for 8.7 years to investigate the association between frailty and onset of cardiovascular disease, independent of subclinical atherosclerotic disease.<br>The analytical sample consisted of 3818 elderly participants (mean age = 76.2 - 5.6 years; female = 64%) with a mean BMI of 27.0 - 4.5 kg/m2 and no cardiovascular disease at baseline.<br>No. of patients with frailty at baseline = 300	CIMT, carotid atheroma plaques, CAC	Initial dates:<br>Frail participants had more moderate or severe carotid plaques (75.6% vs. 62.0%, p = 0.01) and CAC (43.8% vs. 32.6%, p = 0.01) than non-frail participants.<br>Subsequent dates:<br>The cumulative incidence of cardiovascular disease in participants with frailty was higher than in those without frailty (38.9% vs. 25.0%, p < 0.0001).<br>Frailty increased the risk of a CV event during follow-up (OR = 1.35; 95% CI: 1.05-1.74, p = 0.02).	After adjusting the analyses for traditional potential confounders (such as age, gender, and biochemical factors) and, in particular, markers of subclinical atherosclerotic disease, it was found that the association between frailty and cardiovascular disease remained significant.<br>As CAC and carotid plaques are among the strongest predictors of cardiovascular disease, this study further confirms that frailty is an independent risk factor for cardiovascular disease in older adults.
Park et al. 2019[22]	Cross-sectional descriptive study; cohort of 412 adults aged 70-88 years in Busan City, South Korea. Cognitive function was assessed using the Korean Mini-Mental State Examination (K-MMSE), while frailty was assessed using a modified Cardiovascular Health Study frailty index.<br>Data from 231 participants were analysed, with a prevalence of mild cognitive impairment (MCI) of 33.3% and pre-frailty of 55.8%.	CIMT	Analysis of variance (ANOVA) revealed significant differences (p<0.05) in several variables between groups, including weight, body mass index (BMI), blood pressure, grip strength, and carotid intima–media thickness (CIMT). CIMT was significantly correlated with cognitive function and frailty status, with the thickest CIMT observed in the MCI and pre-frailty groups.	The study shows significant associations between cognitive impairment, frailty, and CIMT among older adults. CIMT may serve as a potential marker for cognitive decline and frailty in this population.

Xue et al.[23]	Cross-sectional descriptive study, cohort of 171 patients aged 60-96 years from Tongren Hospital in Beijing. Frailty status was determined using the Fried frailty index.<br>The population was made up of 21.3% frail people, 38.4% pre-frail people, and 40.3% non-frail people.	ABPI, CAVI, CIMT	Frail patients were older, had lower prehension strength (HGS), lower gait speed, lower ABI, and higher CIMT compared to pre-frail and non-frail individuals. ABPI scores were higher in frail patients. Significant inverse linear correlations were found between grip strength, gait speed and CAVI. CAVI was identified as an independent risk factor for frailty (OR: 2.013, 95% CI 1.498-2.703, p,0.001).	This study highlights the association between frailty and various markers of endothelial dysfunction. Of these, the heart–ankle vascular index (CAVI) was identified as an independent risk factor for frailty.
Shiraishi et al. 2022[24]	Cross-sectional descriptive study; cohort of 116 frail Japanese patients in Yokkaichi and Handa Cities, conducted between 2017 and 2019. Frailty was assessed using the Fried frailty index.	CAVI	Long-term sedentary behaviour, exceeding the median value for all participants, was significantly associated ($p<0.05$) with elevated heart–ankle vascular index values, even after adjusting for multiple factors. Even after further adjustment, the association persisted, indicating a significant link between sedentary behaviour and elevated CAVI values.	This study confirms a link between prolonged sedentary behaviour in frail older adults and the degree of arterial stiffness assessed by CAVI.
Yoo et al. [25]	Cross-sectional descriptive study; 236 elderly women belonging to the NAMGARAM-2 cohort in Jinju City, Korea.<br>They were divided into two groups according to their grip strength: 115 of them had normal grip strength, while 121 had low grip strength (<18 kg).	Peripheral arterial tonometry with reactive hyperaemia (RH-PAT)	The endothelial function index in elderly women with low grip strength was found to be worse than in the normal group, with a positive correlation between hand grip strength and endothelial function ($p<0.05$). Endothelial dysfunction, including peripheral arterial tonometry index with reactive hyperaemia, significantly increased the risk of low hand grip strength in a multivariate stepwise analysis.	This study found a correlation between endothelial function and skeletal muscle strength in older women, with lower endothelial function in the low-HGS group and a positive correlation with sarcopenia, suggesting endothelial dysfunction may contribute to sarcopenia.
**Bio-molecular outcomes of endothelial dysfunction**				
**Author/An**	**Study design and setting**	**Assessment of endothelial dysfunction**	**Results and outcome**	**Conclusions**
Marcos-Perez et al. 2018[26]	Cross-sectional descriptive study; cohort of 259 older adults (mean age 73.2+-5.5 years) in the region of Galicia, Spain.<br>Frailty was assessed using the Fried Frailty Index. Thus, of the participants, 88 were frail, 131 pre-frail, and 40 non-frail or robust.<br>-	Lymphocyte subsets*2 , IL6, CRP, sTNF-RII, TNF-α	This study found that frail individuals showed a significant increase ($p<0.05$) in the CD4+/CD8+ ratio and a decrease in the percentage of CD19+ cells. Frailty severity also led to progressive increases in all inflammatory mediator concentrations, with a 70% increase in IL6 and a twofold increase in sTNF-RII in frail subjects compared to non-frail participants. Significant correlations were found between frailty status and inflammatory markers CRP, sTNF-RII, TNFα, IL6, and sTNF-RII.	This study confirms the link between inflammatory molecules and immune activation, supporting the hypothesis that inflammaging is linked to frailty in older adults. Frail subjects show more chronic inflammatory symptoms than expected, particularly in IL6 and sTNF-RII, which are biomarkers that have high accuracy in predicting frailty.

Sayed et al. 2021[27]	Longitudinal descriptive study; cohort of 1001 patients aged 8 to 96 years participating in the Stanford University 1000 Immunomes Project (Stanford 1KIP). For all Stanford 1KIP samples, immune phenotyping was performed at the Stanford HIMC, where peripheral blood samples were processed and analysed using rigorously standardised procedures. Subsequently, deep learning methods of blood immune biomarkers were used to construct a measure for chronic age-related inflammation (iAge).	CXCL9, NO	This study found that iAge in 2010 was predictive of the 2017 frailty score ($p<0.001$). Older people have increased CXCL9 expression and impaired functionality in endothelial progenitor cells compared to younger people. This is evidenced by decreased tubular structure formation, nitric oxide production, and low-density lipoprotein uptake. Silencing the CXCL9 gene in induced pluripotent stem cell-derived endothelial cells significantly improved these aging-related phenotypes.	This study identifies immune biomarkers of ageing and establishes baseline values for chronic systemic inflammation. Researchers created an 'inflammatory clock' using artificial intelligence, highlighting CXCL9 as a key player in cardiovascular pathology, independent of age. Endothelial cells are central to cardiovascular aging, with CXCL9 potentially acting through a positive feedback loop to exacerbate endothelial dysfunction with age.
Álvarez-Sánchez et al.[28]	Cross-sectional descriptive study; 1,211 Spanish men and women aged 65 to 98 years (median age 74 years) from the Toledo Study for Healthy Aging (TSHA) cohort, classified according to Fried's criteria. Thus, of the participants, 50.5% of the patients were frail, 41.3% were pre-frail, and 8.2% were frail.	Hcy and CRP	Hcy was independently associated with frailty (odds ratio [OR] = 1.06; 95% confidence interval [CI]: 1.01-1.12), while hsCRP was independently associated with both pre-frailty (OR = 1.03; 95% CI: 1.01-1.06) and frailty (OR = 1.07; 95% CI: 1.02-1.12). In addition, both markers were positively correlated with the number of Fried criteria met and were independently associated with the criteria of exhaustion (Hcy: OR = 1.03, 95% CI: 1.00-1.06), frailty (hsCRP: OR = 1.03, 95% CI: 1.01-1.05), and low physical activity (hsCRP: OR = 1.04, 95% CI: 1.02-1.06).	Thus, our results highlight the importance of inflammation in age-related physical decline and, in particular, its association with fatigue, decreased strength, and decreased physical activity.
Guillotin et al. 2022[29]	Cross-sectional descriptive study,<br>Cohort of 60 patients suspected of normal pressure hydrocephalus, aged 52 to 92 years, from the University Hospital of Toulouse.<br>Average frailty index = 0.324	Homocysteine (Hcy)	Plasma Hcy level was the only one that correlated with frailty index (adjusted R^2 12% and p-value <0.05). An increase in Hcy is also associated with an increase in age (adjusted R^2 23% and p-value <0.001).	Elevated Hcy levels are suspected as a contributing factor to endothelial dysfunction. In this sense, the association between Hcy and frailty index raises the possibility of association between frailty and endothelial dysfunction.
Alonso-Bouzon et al, 2014[30]	Cross-sectional descriptive study;<br>Cohort of institutionalised Spanish adults living in the community;<br>Age > 65 years;<br>Robust n= 638, Pre-frail n=542, Frail n=107	ADMA	The study found that frail subjects had significantly higher ($p<0.05$) mean ADMA levels compared to pre-frail and non-frail subjects. Frailty was associated with increased risk of ADMA levels, independent of cardiovascular disease (CVD), and was also linked to the presence of atherosclerotic disease.	Endothelial dysfunction, as assessed by ADMA levels, was associated with frailty.<br>This study provides evidence for the role of vascular function from the earliest stages of physical frailty.

Valdiglesias et al. 2018[31]	Cross-sectional descriptive study; cohort of 180 older adults selected from the geriatric outpatient clinic of the Centro di Medicina dell'Invecchiamento (Ce.M.I), Policlinico Agostino Gemelli hospital (Rome, Italy).<br>Exclusion criteria: estimated life < 6 months, inability to walk a distance of 4 m, and unwillingness or inability to give informed consent.<br>All participants included in the study were classified as frail (93 subjects) and non-frail (87 subjects) according to Fried's criteria.	Serum concentrations of neopterin, tryptophan, kynurenine, phenylalanine, tyrosine, nitrite, and C-reactive protein, as well as the ratios of kynurenine/ tryptophan (Kyn/Trp) and phenylalanine/ tyrosine (Phe/Tyr)	A preliminary univariate analysis showed higher levels of neopterin, C-reactive protein, and Kyn/Trp ratio in frail individuals compared to non-frail individuals. However, lower concentrations of tryptophan and nitrite were observed. A multivariate analysis revealed a decrease in nitrite concentrations (p<0.01) and an increase in C-reactive protein concentration (p=0.06) .	This study examined the impact of inflammatory mediators on frailty in older adults. Four parameters, including neopterin, tryptophan, nitrite, and C-reactive protein, showed a strong correlation with frailty. However, nitrite was confirmed as a predictor after multiple regression analysis. Clinical parameters like physical activity, comorbidity, and cognitive impairment were also linked to frailty, suggesting nitrite levels as a potential biomarker.
Mohamad et al. 2018[32]	Cross-sectional descriptive study; cohort of 80 subjects aged 60 years and older were recruited from the outpatient clinic of the Department of Geriatrics at Ain Shams University Hospital, Cairo, Egypt.<br>According to Fried criteria: Robust n=26, Pre-frail n=22, Frail n=32.	IFN-γ, Neopterin, NO	IFN-γ was positively correlated with neopterin (r = 0.576,P = 0.001) and negatively correlated with NO (r = - 0.25, P = 0.03). No significant correlation was detected between neopterin and NO (r = 0.07, P = 0.6).<br>Geriatric depression scale (GDS) was positively correlated with INF-γ (r = 0.24, P = 0.03) and neopterin (r = 0.37, P = 0.001).<br>A one-unit increase in either IFN-γ or neopterin increased the risk of pre-frailty and frailty, while a one-unit increase in NO decreased the risk of frailty.	Higher levels of IFN-γ and neopterin and lower levels of NO were observed in frail and pre-frail subjects compared to healthy subjects.<br>Moreover, INF-γ correlated positively with neopterin and negatively with NO. Increased neopterin levels reflect IFN-γ activity and immune activation; also, neopterin is a marker of GTP-CH1 activity.
Mone et al. 2023[33]	Interventional study; cohort of 40 patients (10 belonging to the control group and 30 divided into three interventional groups) were evaluated over a period of 3 months. All subjects were recruited from Sant'Angelo dei Lombardi Hospital in Avellino, Italy.<br>The three intervention groups received treatment with, respectively: empagliflozin: 10 mg; metformin: 500 mg; and insulin: basal–bolus regimen.<br>Inclusion criteria were age >65 years; previous diagnosis of type 2 DM; frailty objective; and ICFEp.	miRNA (miR)	Assessment of the miR signature of endothelial dysfunction revealed a unique pattern of miRs that were significantly impaired in patients with pFECI compared to the control group and in patients with pFECI before and after treatment with the SGLT2 inhibitor empagliflozin.<br>Thus, three significantly down-regulated miRNAs were miR-126, miR-342-3p, and miR-638, and two miRNAs that were significantly up-regulated in patients with ICFEp compared to healthy controls (P < 0.001) were miR-21 and miR-92. Interestingly, circulating levels of two of the miRNAs (namely miR-21 and miR-92) were significantly (p < 0.001) reduced in ICFEp patients after 3-month treatment with empagliflozin.	This study found five miRs, miR-21, miR-92, miR-126, miR-342-3p, and miR-638, are significantly up/down-regulated in frail heart failure patients compared to healthy ones. These miRs are associated with inflammatory processes, age-related diseases, vascular injury, and endothelial dysfunction. After a 3-month treatment with empagliflozin, miR-21 and miR-92 were down-regulated, suggesting an improvement in endothelial dysfunction. This study also found a complex interaction between miRs and endothelial function, suggesting a specific profile of circulating miRs involved in endothelial function regulation.

			It is worth noting that the mi-Rs mentioned have a demonstrated link in related articles to endothelial dysfunction.	
Mone et al. 2022[34]	Descriptive cross-sectional, cohort study of 325 patients (75 were treated with empagliflozin and 75 were not treated with empagliflozin) evaluated over a 3-month period. All subjects were recruited from Sant'Angelo dei Lombardi Hospital in Avellino, Italy. Inclusion criteria were age >65 years; previous diagnosis of type 2 DM; frailty objective; and ICFEp.	In the case of this article, one of the aims was to highlight the anti-frailty effects of empagliflozin, and thus there was no per se direct quantification of endothelial dysfunction.	Finally, we assessed how many patients in the two study arms had frailty at 3-month follow-up; the empagliflozin-treated group included only 25.3% of patients with frailty (n=19), whereas in the non-empagliflozin group, 73.3% of patients (n=55) had frailty (P<0.001; Figure 2E).	SGLT2 significantly improves cognitive and physical impairment in diabetic and hypertensive patients, most likely by reducing oxidative stress in endothelial cells. Specifically, the dysregulation of mitochondrial capacity has been proposed as a potential root of age-related frailty, and mitochondrial free radicals are crucial players in endothelial dysfunction.
Mello et al. 2022[35]	Descriptive cross-sectional study; cohort of 571 patients belonging to the BRINK (The Brain in Kidney Disease) study[36].<br>Age: 69.3 ± 9.8 years<br>Of the 571 participants:<br>• 433 (75.8%) had eGFRCr <60 ml/min/1.73m^2 —BRC patient group<br>• 138 (24.2%) had eGFRCr >60 ml/min/1.73m^2 —control group.<br>The average SPPB (Short Physical Performance Battery) score for the cohort was 9.4. In total, 222 participants (38.9%) had an SPPB score <10. Stratified by presence or absence, 43.4% (188/433) of participants in the BRC group had SPPB < 10 , compared with 24.6% (34/138) of participants without CKD.	eGFRCr, eGFRCysC, RAC	The study found that lower eGFRCr, lower eGFRCysC, and higher RAC values were associated (p<0.05) with poorer physical performance compared to the control group. For eGFR, lower eGFR was associated with lower SPPB scores. For UACR, higher RAC was associated with lower SPPB scores. In multivariable analysis, eGFRCr was no longer significantly associated with low SPPB scores. However, a higher RAC maintained a significant association with lower SPPB scores when adjusted for eGFRCr and covariates.	The fact that the relationship between albuminuria and physical performance persisted despite extensive adjustment for several risk factors suggests that at least some of the decline in physical performance observed in other similar studies that did not include an assessment of proteinuria may be related to endothelial dysfunction. These findings underscore the importance of exploring albuminuria as a biomarker of reduced physical function in clinical settings, as well as its use in predicting decline in physical performance.

aAIx—aortic augmentation index; ABPI—Ankle–Brachial Pressure Index; aPP—aortic pulse pressure; aPWV—aortic pulse wave velocity; baPWV—brachial–ankle pulse wave velocity; CVD—cardiovascular disease(s); CAC—coronary calcium score; CAVI—cardio–ankle vascular index; cfPWV—carotid–femoral pulse wave velocity; CIMT—intima–media thickness of the carotid artery; CRP—C-reactive protein; TC—total arterial compliance; CD—carotid distensibility coefficient; FMD—flow-mediated dilatation of the brachial artery; Hs-cTnT—high-sensitivity cardiac troponin-T; IL-6—Interleukin-6; miRNA/miR—microRNA; MoCa—Montreal Cognitive Assessment score; NT-proBNP—N-terminal B-type natriuretic propeptide ; UACR—urinary albumin-to-creatinine ratio; GFR—glomerular filtration rate; RHI—Reactive Hyperemia Index; tPA—tissue plasminogen activator; vWF—von Willebrand Factor

Table S3. Quality assessment of articles included in the review

Using the JBI critical appraisal tools for cross-sectional studies									
	**Author/Year**	**Q1**	**Q2**	**Q3**	**Q4**	**Q5**	**Q6**	**Q7**	**Q8**
1	Alonso-Bouzon et al. 2014[30]	Yes	Yes	Yes	Yes	Yes	Yes	Yes	Yes
2	Guillotin et al. 2022[29]	Yes	Yes	Yes	Yes	Yes	Yes	Unsure	Yes
3	Mansur et al. 2015[6]	Yes	Yes	Yes	Yes	Yes	Yes	Yes	Yes
4	McKechnie et al. 2022[19]	Yes	Yes	Yes	Yes	Yes	Yes	Yes	Yes
5	Mello et al. 2022[35]	Yes	Yes	Yes	Yes	Yes	Yes	Yes	Yes
6	Veronese et al. 2017[21]	Yes	Yes	Yes	Yes	Yes	Yes	Yes	Yes
7	Valdiglesias et al. 2018[31]	Yes	Yes	Yes	Yes	Yes	Yes	Yes	Yes
8	Mohamad et al. 2018[32]	Yes	Yes	No	Yes	No	Yes	No	Yes
9	McKechnie et al. 2021[17]	Yes	Yes	Yes	Yes	Yes	Yes	Yes	Yes
10	Lim et al. 2021[18]	Yes	Yes	Yes	Yes	Yes	Yes	Yes	Yes
11	Park et al. 2022[7]	Yes	Yes	Yes	Yes	Yes	Yes	Yes	Yes
12	Yamanashi et al. 2018[14]	Unsure	Yes	Yes	Yes	Yes	Yes	Yes	Yes
13	Park et al. 2019[22]	Yes	Yes	Yes	Yes	Yes	Yes	Yes	Yes
14	Xue et al.[23]	Yes	Yes	Yes	Yes	Yes	Yes	Yes	Yes
15	Álvarez-Bustos et al. 2023[8]	Yes	Yes	Yes	Yes	Yes	Yes	Yes	Yes
16	Nadruz et al. 2016[9]	Yes	Yes	Yes	Yes	Yes	Yes	Yes	Yes
17	Orkaby et al. 2019[15]	Yes	Yes	Yes	Yes	Yes	Yes	Yes	Yes
18	Kannegieter et al. 2016[10]	No	Yes	Yes	Yes	No	No	Yes	Unsure
19	Macêdo et al. 2022[11]	Yes	Yes	Yes	Yes	Yes	No	Yes	Yes
20	Shiraishi et al. 2022[24]	Yes	Yes	Unsure	Yes	Yes	Yes	Yes	Yes
21	Jiang et al. 2023[12]	Yes	Yes	Yes	Yes	Yes	Yes	Yes	Yes
22	Jiang et al. 2022[13]	Yes	Yes	Yes	Yes	Yes	Yes	Yes	Yes
23	Papaioannou et al. 2014[16]	Yes	Yes	Yes	Yes	Yes	Yes	Yes	Yes
24	Marcos-Pérez et al. 2018[26]	Yes	Yes	Yes	Yes	Yes	Yes	Yes	Yes
25	Yoo et al. [25]	Yes	Yes	Yes	Yes	Yes	Yes	Yes	Yes

Using the JBI critical appraisal tools for cohort studies												
	Author/ Year	Q1	Q2	Q3	Q4	Q5	Q6	Q7	Q8	Q9	Q10	Q11

1	Mone 2022[34]	Yes	Yes	Unsure	Yes	Yes	Yes	Yes	Yes	Yes	Yes	Yes
2	Mone 2023[37]	Yes	Yes	Unsure	No	No	Yes	Yes	Yes	Yes	Yes	Yes
3	Sayed 2021[27]	Yes	Yes	Yes	Yes	Yes	Yes	Yes	Unsure	Yes	Yes	Yes

References

1. Page, M.J.; McKenzie, J.E.; Bossuyt, P.M.; Boutron, I.; Hoffmann, T.C.; Mulrow, C.D.; Shamseer, L.; Tetzlaff, J.M.; Akl, E.A.; Brennan, S.E.; et al. The PRISMA 2020 Statement: An Updated Guideline for Reporting Systematic Reviews. *BMJ* **2021**, n71, doi:10.1136/bmj.n71.
2. Amarasekera, A.T.; Chang, D.; Schwarz, P.; Tan, T.C. Does Vascular Endothelial Dysfunction Play a Role in Physical Frailty and Sarcopenia? A Systematic Review. *Age and Ageing* **2021**, *50*, 725–732, doi:10.1093/ageing/afaa237.
3. Des Jarlais, D.C.; Lyles, C.; Crepaz, N.; the TREND Group Improving the Reporting Quality of Nonrandomized Evaluations of Behavioral and Public Health Interventions: The TREND Statement. *Am J Public Health* **2004**, *94*, 361–366, doi:10.2105/AJPH.94.3.361.
4. *JBI Manual for Evidence Synthesis*; Aromataris, E., Munn, Z., Eds.; JBI: Adelaide, Australia, 2020; ISBN 978-0-648-84880-6.
5. Guyatt, G.H.; Oxman, A.D.; Vist, G.E.; Kunz, R.; Falck-Ytter, Y.; Alonso-Coello, P.; Schünemann, H.J. GRADE: An Emerging Consensus on Rating Quality of Evidence and Strength of Recommendations. *BMJ* **2008**, *336*, 924–926, doi:10.1136/bmj.39489.470347.AD.
6. Mansur, H.N.; Lovisi, J.C.M.; Colugnati, F.A.B.; Raposo, N.R.B.; Da Silva Fernandes, N.M.; Bastos, M.G. Association of Frailty with Endothelial Dysfunction and Its Possible Impact on Negative Outcomes in Brazilian Predialysis Patients with Chronic Kidney Disease. *BMC Nephrology* 2015, *16*.
7. Park, W.; Lee, J.; Park, H.-Y.; Park, S.; Park, J. Vascular Function and Frailty in Community-Dwelling Older Individuals. *Artery Research* 2022, *28*, 31–39.
8. Álvarez-Bustos, A.; Carnicero, J.A.; Rodríguez-Sánchez, B.; El-Assar, M.; Rueda, R.; Pereira, S.L.; Sepúlveda-Loyola, W.; Garcia-Garcia, F.J.; Sulo, S.; Rodríguez-Mañas, L. Association Between Pulse Wave Velocity and Frailty, Disability, and Mortality in Community-Dwelling Older Adults. *JACC: Advances* **2023**, *2*, 100423, doi:https://doi.org/10.1016/j.jacadv.2023.100423.
9. Nadruz, W.; Kitzman, D.; Windham, B.G.; Kucharska-Newton, A.; Butler, K.; Palta, P.; Griswold, M.E.; Wagenknecht, L.E.; Heiss, G.; Solomon, S.D.; et al. Cardiovascular Dysfunction and Frailty Among Older Adults in the Community: The ARIC Study. *GERONA* **2016**, glw199, doi:10.1093/gerona/glw199.
10. Kannegieter, L.M.; Tap, L.; Oudshoorn, C.; Van Bruchem-Visser, R.L.; Mattace-Raso, F.U.S. Mobility and Handgrip Strength but Not Aortic Stiffness Are Associated with Frailty in the Elderly. *Journal of Gerontology and Geriatrics* 2016, *64*, 2–8.
11. Macêdo, G.A.D.; Freire, Y.A.; Browne, R.A.V.; Câmara, M.; Cabral, L.L.P.; Schwade, D.; Paulo-Pereira, R.; Silva, R. de M.; Silva, A.M.B.; Farias-Junior, L.F.; et al. Pre-Frailty Phenotype and Arterial Stiffness in Older Adults Free of Cardiovascular Diseases. *Int J Environ Res Public Health* **2022**, *19*.
12. Jiang, X.; Zhou, J.; Yu, C.; Chen, W.; Huang, B.; Chen, Y.; Zhong, L.; Guo, Y.; Geng, Q.; Cai, Y. The Alterations in Multiple Neurophysiological Procedures Are Associated with Frailty Phenotype in Older Adults. *Frontiers in Aging Neuroscience* 2023, *15*.
13. Jiang, X.; Cai, Y.; Wu, X.; Huang, B.; Chen, Y.; Zhong, L.; Gao, X.; Guo, Y.; Zhou, J. The Multiscale Dynamics of Beat-to-Beat Blood Pressure Fluctuation Mediated the Relationship Between Frailty and Arterial Stiffness in Older Adults. *J Gerontol A Biol Sci Med Sci* **2022**, *77*, 2482–2488.
14. Yamanashi, H.; Kulkarni, B.; Edwards, T.; Kinra, S.; Koyamatsu, J.; Nagayoshi, M.; Shimizu, Y.; Maeda, T.; Cox, S.E. Association between Atherosclerosis and Handgrip Strength in Non-hypertensive Populations in India and Japan. *Geriatrics Gerontology Int* **2018**, *18*, 1071–1078, doi:10.1111/ggi.13312.
15. Orkaby, A.R.; Lunetta, K.L.; Sun, F.J.; Driver, J.A.; Benjamin, E.J.; Hamburg, N.M.; Mitchell, G.F.; Vasan, R.S.; Murabito, J.M. Cross-Sectional Association of Frailty and Arterial Stiffness in Community-Dwelling Older Adults: The Framingham Heart Study. *J Gerontol A Biol Sci Med Sci* **2019**, *74*, 373–379.
16. Papaioannou, T.G.; Protogerou, A.D.; Stergiopulos, N.; Vardoulis, O.; Stefanadis, C.; Safar, M.; Blacher, J. Total Arterial Compliance Estimated by a Novel Method and All-Cause Mortality in the Elderly: The PROTEGER Study. *Age* 2014, *36*, 1555–1563.
17. McKechnie, D.G.J.; Papacosta, A.O.; Lennon, L.T.; Ellins, E.A.; Halcox, J.P.J.; Ramsay, S.E.; Whincup, P.H.; Wannamethee, S.G. Subclinical Cardiovascular Disease and Risk of Incident Frailty: The British Regional Heart Study. *Experimental Gerontology* **2021**, *154*, 111522, doi:https://doi.org/10.1016/j.exger.2021.111522.

18. Lim, S.L.; Liu, X.; Gao, Q.; Nyunt, S.Z.; Gong, L.; Lunaria, J.B.; Lam, C.S.; Richards, A.M.; Wee, S.L.; Ling, L.H.; et al. Subclinical Vasculopathy and Skeletal Muscle Metrics in the Singapore Longitudinal Ageing Study. *Aging (Albany NY)* **2021**, *13*, 14768–14784.
19. McKechnie, D.G.J.; Patel, M.; Papacosta, A.O.; Lennon, L.T.; Ellins, E.A.; Halcox, J.P.J.; Ramsay, S.E.; Whincup, P.H.; Wannamethee, S.G. Associations between Inflammation, Coagulation, Cardiac Strain and Injury, and Subclinical Vascular Disease with Frailty in Older Men: A Cross-Sectional Study. *BMC Geriatrics* 2022, *22*.
20. Shaper, A.G.; Pocock, S.J.; Walker, M.; Cohen, N.M.; Wale, C.J.; Thomson, A.G. British Regional Heart Study: Cardiovascular Risk Factors in Middle-Aged Men in 24 Towns. *BMJ* **1981**, *283*, 179–186, doi:10.1136/bmj.283.6285.179.
21. Veronese, N.; Sigeirsdottir, K.; Eiriksdottir, G.; Marques, E.A.; Chalhoub, D.; Phillips, C.L.; Launer, L.J.; Maggi, S.; Gudnason, V.; Harris, T.B. Frailty and Risk of Cardiovascular Diseases in Older Persons: The Age, Gene/Environment Susceptibility-Reykjavik Study. *Rejuvenation Research* 2017, *20*, 517–524.
22. Park, J.; Park, J.-H.; Park, H. Association Between Carotid Artery Intima-Media Thickness and Combinations of Mild Cognitive Impairment and Pre-Frailty in Older Adults. *IJERPH* **2019**, *16*, 2978, doi:10.3390/ijerph16162978.
23. Xue, Q.; Qin, M.-Z.; Jia, J.; Liu, J.-P.; Wang, Y. Association between Frailty and the Cardio-Ankle Vascular Index. *Clin Interv Aging* **2019**, *14*, 735–742, doi:10.2147/CIA.S195109.
24. Shiraishi, N.; Suzuki, Y.; Kuromatsu, I.; Komiya, H.; Kuzuya, M. Sedentary Behavior Is Associated with Arteriosclerosis in Frail Older Adults. *Nagoya J Med Sci* **2022**, *84*, 91–100.
25. Yoo, J.-I.; Kim, M.-J.; Na, J.-B.; Chun, Y.-H.; Park, Y.-J.; Park, Y.; Hah, Y.-S.; Ha, Y.-C.; Park, K.S. Relationship between Endothelial Function and Skeletal Muscle Strength in Community Dwelling Elderly Women. *J Cachexia Sarcopenia Muscle* **2018**, *9*, 1034–1041, doi:10.1002/jcsm.12340.
26. Marcos-Pérez, D.; Sánchez-Flores, M.; Maseda, A.; Lorenzo-López, L.; Millán-Calenti, J.C.; Gostner, J.M.; Fuchs, D.; Pásaro, E.; Laffon, B.; Valdiglesias, V. Frailty in Older Adults Is Associated With Plasma Concentrations of Inflammatory Mediators but Not With Lymphocyte Subpopulations. *Front. Immunol.* **2018**, *9*, 1056, doi:10.3389/fimmu.2018.01056.
27. Sayed, N.; Huang, Y.; Nguyen, K.; Krejciova-Rajaniemi, Z.; Grawe, A.P.; Gao, T.; Tibshirani, R.; Hastie, T.; Alpert, A.; Cui, L.; et al. An Inflammatory Aging Clock (iAge) Based on Deep Learning Tracks Multimorbidity, Immunosenescence, Frailty and Cardiovascular Aging. *Nat Aging* **2021**, *1*, 598–615.
28. Álvarez-Sánchez, N.; Álvarez-Ríos, A.I.; Guerrero, J.M.; García-García, F.J.; Rodríguez-Mañas, L.; Cruz-Chamorro, I.; Lardone, P.J.; Carrillo-Vico, A. Homocysteine and C-Reactive Protein Levels Are Associated With Frailty in Older Spaniards: The Toledo Study for Healthy Aging. *The Journals of Gerontology: Series A* **2020**, *75*, 1488–1494, doi:10.1093/gerona/glz168.
29. Guillotin, S.; Vallet, A.; Lorthois, S.; Cestac, P.; Schmidt, E.; Delcourt, N. Association Between Homocysteine, Frailty and Biomechanical Response of the CNS in NPH-Suspected Patients. *Journals of Gerontology - Series A Biological Sciences and Medical Sciences* 2022, *77*, 1335–1343.
30. Alonso-Bouzón, C.; Carcaillon, L.; García-García, F.J.; Amor-Andrés, M.S.; El Assar, M.; Rodríguez-Mañas, L. Association between Endothelial Dysfunction and Frailty: The Toledo Study for Healthy Aging. *Age* 2014, *36*, 495–505.
31. Valdiglesias, V.; Marcos-Pérez, D.; Lorenzi, M.; Onder, G.; Gostner, J.M.; Strasser, B.; Fuchs, D.; Bonassi, S. Immunological Alterations in Frail Older Adults: A Cross Sectional Study. *Experimental Gerontology* **2018**, *112*, 119–126, doi:https://doi.org/10.1016/j.exger.2018.09.010.
32. Mohamad, M.; Ebeid, S.; Khater, M.S.; Alsadany, M. Interferon- Gamma- Inducible Guanosine Triphosphate Cyclohydrolase 1 (GTP-CH1) Pathway Is Associated with Frailty in Egyptian Elderly. *Reports of Biochemistry and Molecular Biology* 2017, *7*, 52–58.
33. Mone, P.; Lombardi, A.; Kansakar, U.; Varzideh, F.; Jankauskas, S.S.; Pansini, A.; Marzocco, S.; De Gennaro, S.; Famiglietti, M.; Macina, G.; et al. Empagliflozin Improves the MicroRNA Signature of Endothelial Dysfunction in Patients with Heart Failure with Preserved Ejection Fraction and Diabetes. *J Pharmacol Exp Ther* **2022**, *384*, 116–122.
34. Mone, P.; Varzideh, F.; Jankauskas, S.S.; Pansini, A.; Lombardi, A.; Frullone, S.; Santulli, G. SGLT2 Inhibition via Empagliflozin Improves Endothelial Function and Reduces Mitochondrial Oxidative Stress: Insights from Frail Hypertensive and Diabetic Patients. *Hypertension* **2022**, *79*, 1633–1643, doi:10.1161/HYPERTENSIONAHA.122.19586.

35. Mello, R.; Johansen, K.L.; Murray, A.; Davey, C.; Hart, A. Estimated GFR, Albuminuria, and Physical Function: The Brain in Kidney Disease (BRINK) Cohort Study. *Kidney Medicine* **2022**, *4*, 100531, doi:https://doi.org/10.1016/j.xkme.2022.100531.
36. Murray, A.M.; Bell, E.J.; Tupper, D.E.; Davey, C.S.; Pederson, S.L.; Amiot, E.M.; Miley, K.M.; McPherson, L.; Heubner, B.M.; Gilbertson, D.T.; et al. The Brain in Kidney Disease (BRINK) Cohort Study: Design and Baseline Cognitive Function. *American Journal of Kidney Diseases* **2016**, *67*, 593–600, doi:10.1053/j.ajkd.2015.11.008.
37. Mone, P.; Lombardi, A.; Kansakar, U.; Varzideh, F.; Jankauskas, S.S.; Pansini, A.; Marzocco, S.; De Gennaro, S.; Famiglietti, M.; Macina, G.; et al. Empagliflozin Improves the MicroRNA Signature of Endothelial Dysfunction in Patients with Heart Failure with Preserved Ejection Fraction and Diabetes. *Journal of Pharmacology and Experimental Therapeutics* 2023, *384*, 116–122.
